# Impacts of intracranial pressure monitoring on mortality and length of stay in severe traumatic brain injury: a systematic review and meta-analysis

**DOI:** 10.1007/s00068-026-03302-5

**Published:** 2026-08-12

**Authors:** Michael Merakis, NakHyun Kim, Gina Velli, Zsolt J. Balogh

**Affiliations:** 1 Department of Neurosurgery, John Hunter Hospital, Newcastle, NSW Australia; 2 Department of Trauma Surgery, John Hunter Hospital, Lookout Road, New Lambton Heights, Newcastle, NSW 2305 Australia; 3 College of Health, Medicine and Wellbeing, University of Newcastle, Newcastle, NSW Australia; 4 Department of Neurosurgery, Princess Alexandra Hospital, Brisbane, QLD Australia; 5 Injury and Trauma Research Program, Hunter Medical Research Institute, Newcastle, NSW Australia; 6 PAH Library and Knowledge Centre, Princess Alexandra Hospital, Brisbane, QLD Australia

**Keywords:** Intracranial pressure monitoring, Mortality, Outcome, Traumatic brain injury, Systematic review

## Abstract

**Purpose:**

This systematic review and meta-analysis examined the association between intracranial pressure monitoring (ICPm) and mortality in patients with severe traumatic brain injury (sTBI), with additional focus on functional neurological outcomes and intensive care unit (ICU) and hospital length of stay (LOS).

**Methods:**

This review followed Preferred Reporting Items for Systematic Reviews and Meta-Analyses (PRISMA) guidelines and was registered with PROSPERO (CRD42025643607). MEDLINE, Embase, and the Cochrane Central Register of Controlled Trials were searched through August 1, 2025, for studies comparing ICP-monitored and non-ICP-monitored patients with traumatic brain injury. Eligible study designs included randomized controlled trials, prospective observational cohort studies, retrospective observational cohort studies, registry-based cohort studies, and case-control studies. Study quality was assessed using the Strengthening the Reporting of Observational Studies in Epidemiology checklist, Risk of Bias 2.0 for randomized trials, and the Oxford Centre for Evidence-Based Medicine Levels of Evidence framework.

**Results:**

Thirty-one studies met inclusion criteria. The mortality meta-analysis included 121,799 patients. Pooled crude analysis showed lower mortality in ICP-monitored patients than in non-monitored patients (OR 0.79, 95% CI 0.68–0.91, p = 0.002), with high heterogeneity (I²=92%). Across studies with extractable group-level mortality percentages, median mortality was 28.6% in ICP-monitored patients and 33.2% in non-monitored patients. Functional neurological outcomes were inconsistently reported and did not show a consistent benefit with ICPm. ICU and hospital LOS were generally longer in ICP-monitored patients. Ten studies reported complication data, most commonly infection, hemorrhagic events, and mechanical or device-related complications; reporting was heterogeneous and precluded pooled analysis.

**Conclusions:**

ICPm was associated with lower crude mortality in sTBI; however, substantial heterogeneity and residual confounding limit causal interpretation. Functional outcomes did not show consistent benefit. Complication reporting was inconsistent, but reported events included infection, hemorrhage, and device-related complications. Future studies should standardize functional outcome reporting and adjust for key confounders.

**Level of evidence:**

Systematic review and meta-analysis; Level III.

**Supplementary Information:**

The online version contains supplementary material available at 10.1007/s00068-026-03302-5.

## Background

Severe traumatic brain injury (sTBI) is devastating and leaves survivors with profound, lasting disabilities [1–3]. Traumatic brain injury (TBI) has an enormous burden of disease financially, socially, emotionally, and physically [4, 5]. TBI is common, with some estimates placing the global prevalence as high as 55 million and the annual incidence around 369 per 100,000 people [6]. Mortality rates after sTBI differ across the literature, although most studies report rates around 30% [7]. TBI contributes significantly to the global economic burden of disease [8, 9]. 

Acute TBI care centers on prevention of secondary brain injury to optimize outcomes. Persistently raised intracranial pressure can worsen secondary neurological injury by reducing cerebral perfusion and oxygenation and by contributing to blood–brain barrier disruption, neuroinflammation, and excitotoxic injury [10, 11]. Invasive intracranial pressure (ICP) monitors are inserted through the skull into the intracranial compartment to provide continuous ICP measurement. Several types of monitoring devices are available, the most common are intraparenchymal strain gauge devices and ventriculostomy catheters. ICP monitoring (ICPm) may be particularly useful in sTBI when depressed consciousness, sedation, or extracranial injuries limit serial neurological assessment.

An emerging body of literature describes the utilization and effectiveness of ICP monitoring in patients with sTBI. Previous reviews have primarily focused on mortality, with limited reporting of detailed data on functional neurological outcomes [8, 12, 13]. Some previous systematic reviews demonstrated inconsistency in study selection, omitting studies that were present in other reviews. Several additional comparative studies have been published since earlier systematic reviews and meta-analyses [12]. Large-scale prospective cohort studies have demonstrated variation in the utilization and application of ICPm, with variable outcomes regarding mortality and neurological recovery [13–15]. 

The current Brain Trauma Foundation 4th edition guidelines recommend management of severe TBI using information from ICP monitoring to reduce in-hospital and 2-week mortality [16]. The classic BTF patient-selection criteria, published in the 3rd edition, advised ICP monitoring in salvageable patients with severe TBI, defined as GCS 3–8 after resuscitation, with an abnormal CT scan showing hematoma, contusion, swelling, herniation, or compressed basal cisterns. In patients with severe TBI and a normal CT scan, ICP monitoring was advised when at least two of the following were present: age older than 40 years, unilateral or bilateral motor posturing, or systolic blood pressure < 90 mmHg [17]. These criteria were reproduced in the 4th edition as prior-edition recommendations, but were not carried forward as formal recommendations because the supporting evidence did not meet current guideline standards [16]. 

Therefore, the aim of this review is to include the latest data to evaluate the impact of ICPm on mortality in sTBI, with a focus on functional neurological outcomes and hospital or intensive care unit (ICU) length of stay (LOS).

## Methods

This review followed the Preferred Reporting Items for Systematic Reviews and Meta-Analyses (PRISMA) guidelines [18]. The completed PRISMA 2020 checklist is provided in Supplemental Digital Content 1 (SDC 1). The review protocol was registered with the International Prospective Register of Systematic Reviews (PROSPERO; registration number: CRD42025643607) [19]. Protocol deviations were reviewed at revision. The search was performed without date restrictions rather than commencing in 2013, as originally registered, to avoid excluding earlier comparative ICP monitoring studies relevant to the review question. GRADE assessment was planned in the protocol but was not performed because the evidence base was dominated by heterogeneous observational studies, secondary outcomes were not pooled, and the primary pooled analysis was limited to crude mortality data; instead, evidence appraisal was reported using OCEBM levels of evidence, STROBE reporting assessment and structured confounding-control extraction. For the included randomized controlled trial, RoB 2.0 was assessed across the relevant outcome domains extracted in this review, including mortality, GOS-E, and ICU length of stay.

### Search strategy

A systematic search of databases, MEDLINE (PubMed), Embase, and the Cochrane Central Register of Controlled Trials, was performed through August 1, 2025. The search was not limited by language or date. Animal studies were excluded. Key search terms included synonyms and controlled subject headings relating to intracranial pressure, intracranial hypertension, ICP monitor, intracranial pressure monitoring, TBI, traumatic brain injury, brain injury, and craniocerebral trauma. The full search strategy is provided in Supplemental Digital Content 2 (SDC 2), Search strategy. The search was not extended to include registers and grey literature. The search was translated and extended using an integrated search interface [20]. In addition to MEDLINE, Embase, and CENTRAL, supplementary searches were conducted in Google Scholar and ISI through the integrated search interface, with additional records identified through reference screening and forward citation searching. Papers that met the inclusion criteria were further assessed for seed references and forward citation searched in line with the Terminology, Application, and Reporting of Citation Searching (TARCiS) statement [21]. 

### Inclusion criteria

Study types eligible for inclusion were randomized clinical trials, prospective observational cohort studies, retrospective observational cohort studies, registry-based cohort studies, and case-control studies. Non-original research formats, such as case reports and reviews were excluded. Studies suitable for inclusion compared hospitalized patients treated with and without ICPm who suffered a traumatic brain injury and reported on the primary outcome of mortality. Studies were not excluded solely because they included pediatric or mixed-age cohorts, provided they otherwise met the review criteria. The registered protocol specified adult patients, defined as age ≥ 18 years; however, this age restriction was not applied during final study selection, and pediatric or mixed-age cohorts were retained when they otherwise met the review criteria. This deviation was made to avoid excluding major comparative ICP monitoring studies and to present the full comparative evidence base. Retracted papers were deemed ineligible for inclusion. Publications with minor errata, unrelated to study quality, were not excluded. Further details can be found in the registered study protocol.

### Screening

Two reviewers independently evaluated citations and selected eligible studies based on inclusion criteria, where disagreement persisted after discussion, senior author adjudication was sought. Where overlapping datasets were identified, the more relevant parent comparative cohort was retained, and the less relevant overlapping or device-subset analysis was excluded. Screening was conducted using EndNote 21 (RRID: SCR_014001) and Microsoft Excel (RRID: SCR_016137) [22, 23]. 

### Data extraction

Key study characteristics were extracted by two authors into a Microsoft Excel spreadsheet and included author, publication year, country, design, and sample size. Treatment variables included the use of ICPm, type of monitor used, and duration of monitoring. Patient outcome measures included mortality rates, functional outcome measures such as the Glasgow Outcome Scale (GOS), Glasgow Outcome Scale–Extended (GOS-E/GOSE), and Functional Independence Measure (FIM), ICU or hospital LOS, crude or adjusted odds ratios (ORs) for mortality, and confounding factors. Some included mortality figures were not explicitly reported in the original studies but were derived from extractable 2 × 2 contingency tables. Overlapping data were identified across publications using the same or partially overlapping datasets; where overlap was identified, the more relevant parent comparative cohort was retained, and the less relevant subset or device-comparator analysis was excluded. Extracted data was cross checked amongst authors extracting the data and discrepancies were resolved by consensus or senior author adjudication where required.

### Reporting quality and risk-of-bias assessment

Included observational studies were assessed for reporting quality using the Strengthening the Reporting of Observational Studies in Epidemiology (STROBE) checklist [24]. RCTs were assessed using Risk of Bias 2.0 (RoB 2.0) [25]. Two reviewers worked collaboratively to assess methodological quality, STROBE assessments and RoB 2.0 judgments were recorded in tabulated format in Microsoft Word, with RoB 2.0 assessment conducted according to the Cochrane RoB 2.0 tool; disagreements were resolved by consensus, with senior author adjudication where required. The Newcastle-Ottawa Scale was not used because the included observational evidence comprised heterogeneous prospective, retrospective, registry-based, propensity-adjusted, and multilevel cohort designs; instead, reporting completeness was assessed using STROBE, level of evidence was assessed using the OCEBM framework, and confounding control was extracted separately in a structured supplementary table.

### Synthesis and analysis

Study characteristics, population characteristics, monitoring practices, and outcomes were summarized descriptively. Where meta-analysis was not appropriate because of heterogeneity in outcome definitions, reporting format, or timing of assessment, findings were synthesized qualitatively in accordance with Synthesis Without Meta-analysis (SWiM) guidance [26]. The number of studies reporting each outcome was presented as a count and percentage of the total included studies. Secondary outcomes, including functional neurological outcomes, ICU and hospital LOS, and complications, were synthesized descriptively and tabulated by study. Functional outcomes were grouped according to the outcome measure used by the original study, including GOS, GOS-E/GOSE, FIM, favorable or unfavorable outcome, and discharge disposition. LOS and complication outcomes were reported in the format provided by the original studies. Meta-analysis of secondary outcomes was not performed because functional outcomes, LOS, and complications were reported using heterogeneous definitions, timepoints, scales, and summary statistics, which prevented clinically meaningful pooled estimates.

Mortality was the primary outcome for quantitative synthesis. Crude odds ratios (ORs) for mortality were calculated from extractable group-level mortality data where not directly reported by the original study. Mortality proportions were summarized across studies using median and interquartile range (IQR), and absolute differences between ICP-monitored and non-ICP-monitored groups were calculated descriptively. Mortality was pooled using a random-effects Mantel–Haenszel meta-analysis of crude odds ratios. Study weights in the mortality meta-analysis were generated statistically within the random-effects Mantel–Haenszel model according to study size, event counts, and between-study variance. No manual quality-based weighting was applied. This approach was selected because mortality was a dichotomous outcome with extractable group-level event counts, and because substantial clinical and methodological heterogeneity was expected across studies. Statistical heterogeneity was quantified using the I² statistic, with I² >60% considered high. Results were presented as a forest plot. Because study design was an important source of methodological heterogeneity, a study-design subgroup analysis was performed separating the randomized controlled trial from observational studies. Mortality was also summarized qualitatively because of high statistical heterogeneity and the predominance of non-randomized evidence.

### Level of evidence

The level of evidence in the reviewed studies was evaluated and graded using the ‘Oxford Levels of Evidence’ framework, developed by the Oxford Centre for Evidence-Based Medicine (OCEBM) Levels of Evidence Working Group (2011) [27]. The OCEBM grading system classifies evidence from Level 1, representing the strongest evidence, to Level 5, representing the weakest evidence.

## Results

**Fig. 1:**
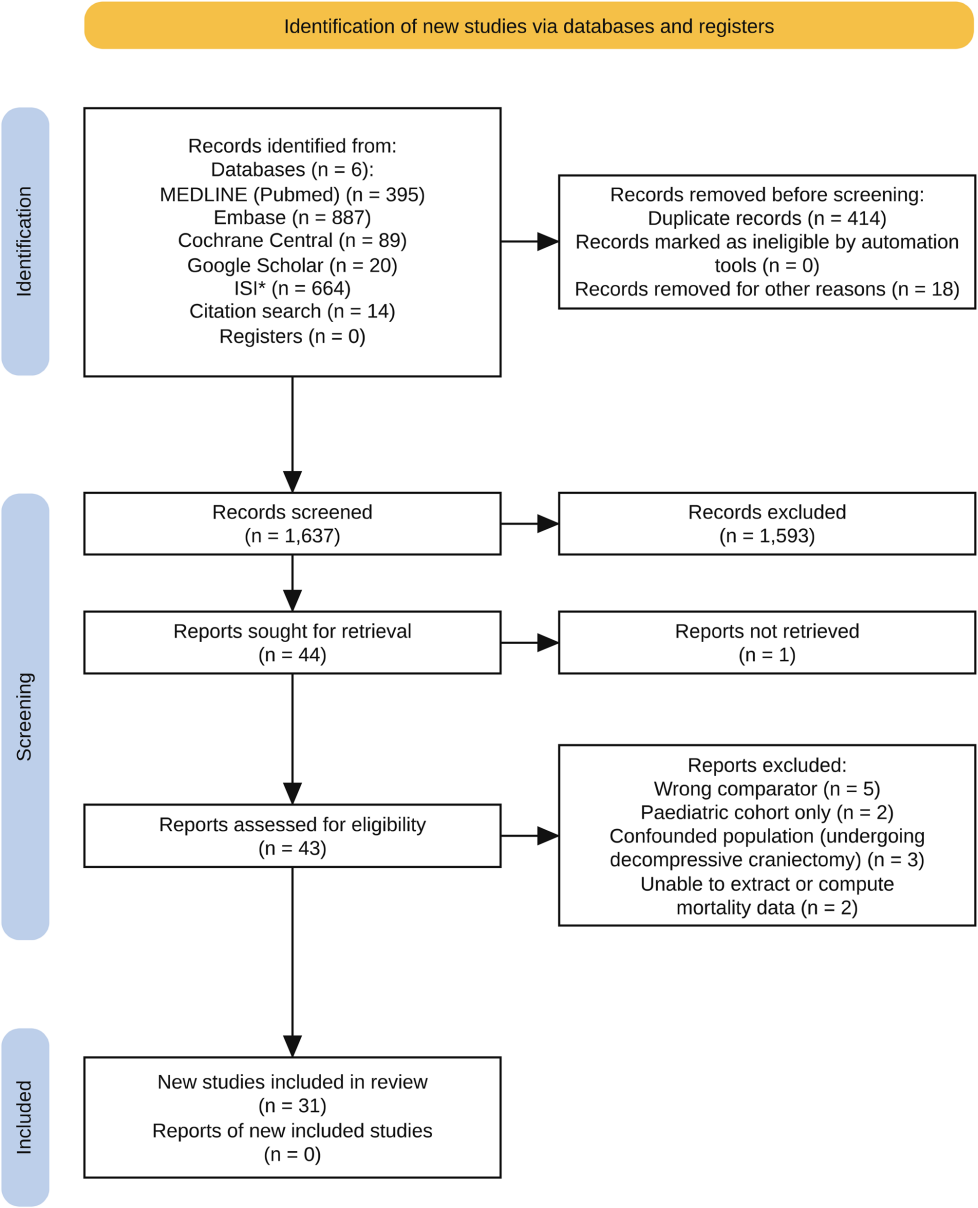
PRISMA flow diagram of study identification, screening, eligibility assessment, and inclusion

The database and citation search identified 2,069 records: MEDLINE (*n* = 395), Embase (*n* = 887), Cochrane Central (*n* = 89), Google Scholar (*n* = 20), ISI (*n* = 664), and reference screening and citation searching (*n* = 14). After removal of 414 duplicate records and 18 records removed for other reasons, 1,637 records underwent title and abstract screening. Of these, 1,593 records were excluded, leaving 44 reports sought for retrieval. One report could not be retrieved, and 43 reports were assessed for eligibility. Twelve reports were excluded: wrong comparator (*n* = 5), pediatric cohort only (*n* = 2), confounded population undergoing decompressive craniectomy (*n* = 3), and inability to extract or compute mortality data (*n* = 2). Full-text articles were further assessed for errata, retractions, errors, and omissions. One included study had a minor erratum, which did not affect study validity or eligibility [28, 29]. In total, 31 studies met the inclusion criteria and were included in the review. The study-selection process is summarized in the PRISMA flow diagram (Fig. 1). Studies excluded after full-text assessment are listed in Supplemental Digital Content 5 (SDC 5).

### Study characteristics

In total, 31 studies met the inclusion criteria and are summarized in Table 1. Of these, studies contributing extractable mortality data to the meta-analysis included 121,799 patients with severe traumatic brain injury [10, 13–15, 29–55]. Severe TBI was most commonly defined by a Glasgow Coma Scale (GCS) score of 3–8 or < 9, although inclusion criteria varied across studies, with some cohorts restricted by age, CT findings, operative subgroup, registry criteria, or guideline-based eligibility. Included studies were published between 2000 and 2025 and included one randomized controlled trial [31], 13 prospective observational studies [10, 13–15, 30, 34, 38–40, 42, 43, 49, 51], and 17 retrospective cohort or registry-based studies [29, 32, 33, 35–37, 41, 44–48, 50, 52–55]. Study settings were geographically diverse, with data from North America, Europe, Asia, the Middle East, and one international cohort spanning 42 countries. Sample sizes ranged from 61 to 36,929 patients. Monitoring modality was variably reported: some studies used intraparenchymal monitors or intraventricular catheters exclusively [31, 33, 36, 37, 40, 43, 48, 49, 51], whereas others grouped multiple invasive monitoring modalities together or did not specify device type [10, 13–15, 29, 32, 34, 35, 38, 39, 41, 42, 44–47, 50, 52–55]. Detailed study characteristics and population demographics are presented in Supplemental Digital Content 6a (SDC 6a).

**Table 1:** Characteristics of included studies

Study, period		Location, Database, Study type	*n*	Monitor assessed	Inclusion criteria
Agrawal et al. 2015	2010–2012	India; registry; prospective cohort with PS adjustment	1,345	Intraparenchymal	GCS ≤ 8 meeting BTF ICPm criteria; excluded death < 24 h
Aiolfi et al. 2017	2013–2014	USA; TQIP; retrospective cohort	13,188	Mixed	BTF ICPm-eligible severe blunt TBI; excluded major extracranial injury/transfers
Alali et al. 2013	2009–2011	USA/Canada; ACS TQIP; retrospective cohort with multilevel adjustment	10,628	Mixed	GCS ≤ 8 with acute intracranial lesion; excluded penetrating/polytrauma/early deaths
Alali et al. 2015	2010–2012	USA; ACS TQIP; retrospective pediatric cohort with PSM	1,705	Mixed	Age ≤ 16 y, GCS ≤ 8; excluded penetrating injury/polytrauma
Al-Anazi et al. 2004	1992–2002	Saudi Arabia; none; retrospective cohort	178	Intraventricular	GCS ≤ 8 with ICU admission and CT-confirmed injury
Alkhoury et al. 2014	2001–2006	USA; NTDB; retrospective pediatric cohort	3,107	Mixed	Age < 17 y, GCS < 9, ISS > 9, ICU admission, CT evidence of TBI
Al Saiegh et al. 2020	2000–2017	USA; statewide trauma registry; retrospective cohort	36,929	Mixed	Age > 18 y, admission GCS < 9; excluded dead on arrival
Biersteker et al. 2012	2008–2009	Netherlands; POCON; prospective cohort	265	Mixed	Age ≥ 16 y meeting BTF ICPm criteria; excluded delayed admission/gunshot injury
Chesnut et al. 2012	2008–2011	Bolivia/Ecuador; BEST: TRIP; RCT	324	Intraparenchymal	GCS 3–8 or early deterioration; ICU admission; excluded unsurvivable injury
Dawes et al. 2015	2009–2010	USA; none; prospective cohort with PSM	822	Mixed	ED GCS ≤ 8 with abnormal intracranial CT; excluded dead on arrival
Farahvar et al. 2012	2000–2009	USA; BTF TBI-trac; prospective cohort	1,307	Mixed	GCS < 9 for ≥ 6 h; ICP-lowering therapy in first 2 days
Foote et al. 2022	2015–2020	USA; none; retrospective cohort	123	Mixed	GCS ≤ 8 with abnormal CT; consecutive trauma admissions
Fu et al. 2020	2012–2013	China; Huashan TBI database; retrospective cohort with PSM	651	Mixed	Age > 17 y, GCS ≤ 13, cerebral contusion > 20 mL, admission ≤ 24 h
Gao et al. 2013	-	China; none; retrospective operative cohort	63	Intraventricular	Traumatic bifrontal contusions requiring surgery; mixed GCS severity
Griesdale et al. 2010	2000–2006	Canada; none; retrospective cohort with PS adjustment	171	Intraventricular	GCS ≤ 8, ICU admission; excluded early death/rapid neurological improvement
Haddad et al. 2011	2001–2008	Saudi Arabia; none; retrospective ICU cohort	477	Intraventricular	Age ≥ 18 y, GCS 3–8, ICU admission; excluded brain death on admission
Jitchanvichai et al. 2024	2013–2018	Thailand; none; retrospective cohort with PSM	849	Intraparenchymal	Age ≥ 15 y, post-resuscitation GCS ≤ 8, preoperative CT available
Kostić et al. 2011	2008–2010	Serbia; none; prospective quasi-randomized study	61	Mixed	GCS ≤ 8 or abnormal CT with high risk of intracranial hypertension
Lane et al. 2000	1989, 1991–1995	Canada; Ontario Trauma Registry; retrospective cohort	5,507	Mixed	ISS > 12 and head MAIS > 3 from Ontario Trauma Registry
Lele et al. 2019	2012–2014	India; CHIRAG; prospective cohort with IPW	200	Intraparenchymal	Age ≥ 18 y, post-resuscitation GCS < 8, abnormal CT, MV > 48 h
Mauritz et al. 2008	1998–2004	Austria; ICU registry; prospective cohort	1,856	Mixed	GCS < 9 or pre-sedation GCS < 9, AIS head > 2, ICU admission
Nattino et al. 2023	2014–2019	Italy/Hungary; CREACTIVE; prospective cohort with PSM	1,448	Mixed	Adults meeting BTF ICPm criteria; ICU admission ≤ 2 days
Peled et al. 2025	2015–2021	Israel; National Trauma Registry; retrospective cohort	2,202	Mixed	GCS 3–8 with AIS head ≥ 3; excluded ED deaths/missing ICP data
Robba et al. 2021	2018–2019	International; SYNAPSE-ICU; prospective cohort with IPW	2,367	Mixed	Adults with acute brain injury and impaired consciousness admitted to ICU
Schupper et al. 2019	2010–2014	USA; NTDB; retrospective cohort with PSM	30,710	Mixed	GCS 3–8; excluded major extracranial injury, short LOS, transfers
Shafi et al. 2008	1994–2001	USA; NTDB; retrospective cohort	1,646	Mixed	Age 20–50 y, GCS ≤ 8 with abnormal CT; ICU LOS ≥ 3 days
Suehiro et al. 2017	2009–2011	Japan; JNTDB; retrospective cohort	1,091	Mixed	GCS ≤ 8, early deterioration, or post-craniotomy traumatic hematoma
Talving et al. 2013	2010–2011	USA; none; prospective cohort with PS adjustment	216	Mixed	Age ≥ 18 y, GCS ≤ 8, head AIS ≥ 3, BTF ICPm-eligible
Yang et al. 2022	2014–2017	China; CENTER-TBI China; prospective cohort with PSM	2,029	Mixed	GCS ≤ 8 admitted to ICU; excluded missing GCS/ICP/survival data
You et al. 2016	2008–2014	China; none; prospective cohort	166	Intraventricular	Age ≥ 65 y, GCS < 9, abnormal CT, admission ≤ 24 h
Zeng et al. 2013	2010–2012	China; none; prospective controlled cohort	168	Intraventricular	Age 15–70 y, GCS < 13, normal baseline renal function

NB: Values in the n column refer to patients included in the comparative ICP-monitored versus non-ICP-monitored analysis used for this review, where extractable. Population descriptors are summarized as reported in the original studies. Monitor type is listed as reported; “Mixed” indicates that multiple invasive ICP monitoring modalities were grouped or that the specific device type was not consistently reported

Abbreviations: AIS, Abbreviated Injury Scale; BTF, Brain Trauma Foundation; CT, computed tomography; ED, emergency department; EVD, external ventricular drain; GCS, Glasgow Coma Scale; ICPm, intracranial pressure monitoring; ICU, intensive care unit; ICHT, intracranial hypertension; IP, intraparenchymal; ISS, Injury Severity Score; IV, intraventricular; LOS, length of stay; MAIS, maximum Abbreviated Injury Scale; MV, mechanical ventilation; PSM, propensity score matching; RCT, randomized controlled trial; TBI, traumatic brain injury; TQIP, Trauma Quality Improvement Program

### Risk of bias within studies

Study quality and reporting were assessed using the Oxford Centre for Evidence-Based Medicine levels of evidence [27], the STROBE checklist for observational studies [24], and the Risk of Bias 2.0 tool for the included randomized trial [25]. Most included evidence was observational, with one randomized controlled trial [31], 13 prospective observational studies [10, 13–15, 30, 34, 38–40, 42, 43, 49, 51], and 17 retrospective cohort or registry-based studies [29, 32, 33, 35–37, 41, 44–48, 50, 52–55]. STROBE reporting quality was rated as good in 24 observational studies (80%) [10, 13–15, 29, 32, 35–43, 45, 46, 48, 49, 51–55], fair in five studies (17%) [30, 34, 44, 47, 50] and limited in one study (3%) [33]. The most frequent reporting limitations were absent funding statements, incomplete reporting of bias or data-source methods, and limited discussion of generalizability. Confounding control varied substantially between studies; age and GCS were the most commonly adjusted variables, whereas pupillary response, CT-defined injury severity, extracranial injury burden, and physiological severity were less consistently incorporated. The single randomized controlled trial, Chesnut et al. 2012 [31], was assessed using the RoB 2.0 tool and was judged to have low risk of bias across all domains. Methodological quality, STROBE reporting assessment, RoB 2.0 assessment, and confounding control are summarized in Supplemental Digital Content 3 (SDC 3) and Supplemental Digital Content 4 (SDC 4).

### Mortality

**Fig. 2:**
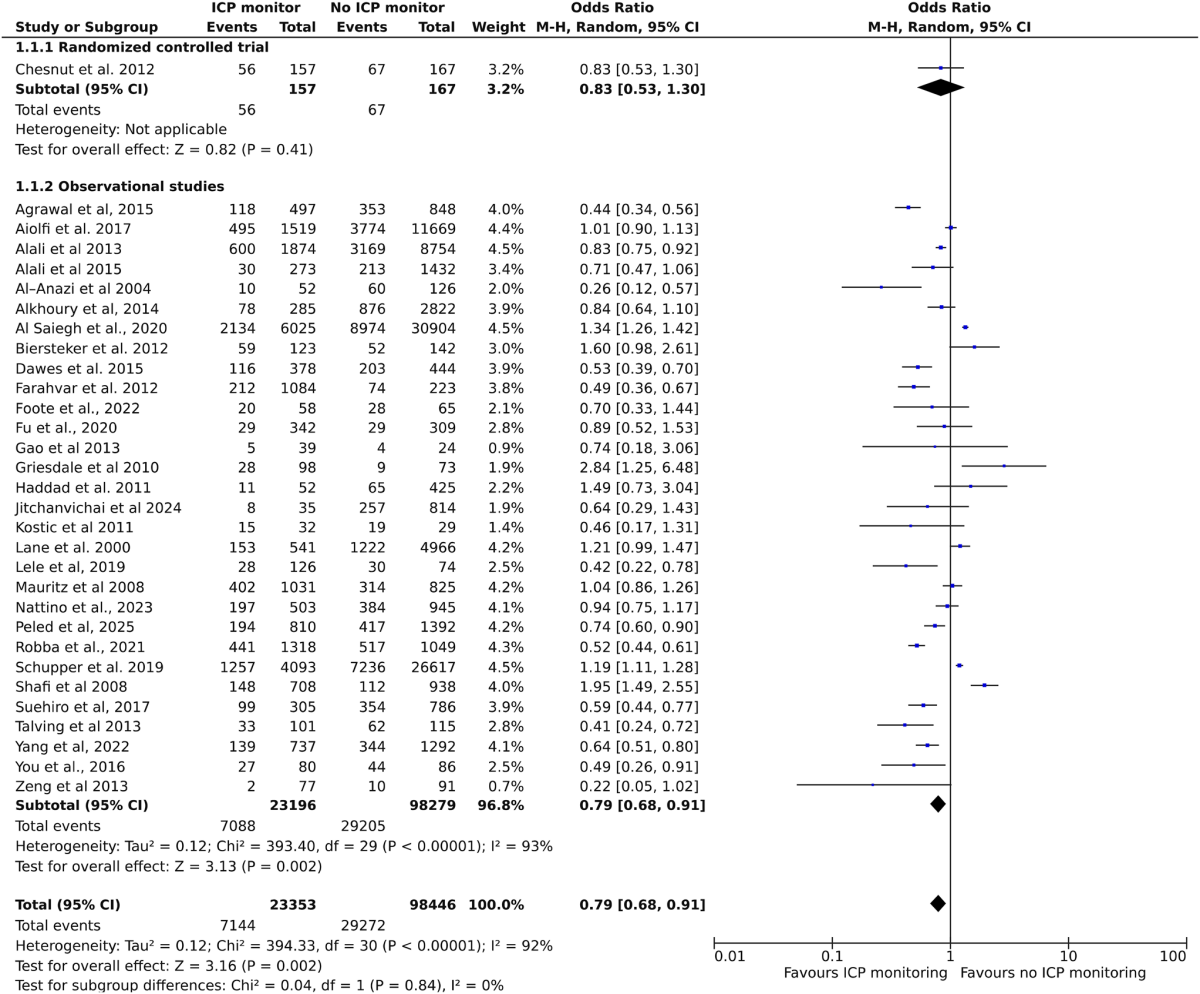
Forest plot of crude mortality odds ratios for ICP-monitored versus non-ICP-monitored patients with traumatic brain injury, stratified by study design. Odds ratios less than 1 favour ICP monitoring

Mortality outcomes were reported across all included studies and are summarized in Table 2. The pooled crude analysis demonstrated lower mortality in ICP-monitored patients compared with non-monitored patients (OR 0.79, 95% CI 0.68–0.91, *p* = 0.002). Statistical heterogeneity was high (I²=92%), consistent with substantial clinical and methodological variation across the included studies (Fig. 2). In study-design subgroup analysis, the single randomized controlled trial showed no significant mortality benefit with ICP-guided care (OR 0.83, 95% CI 0.53–1.30), while pooled observational studies showed lower crude mortality with ICPm (OR 0.79, 95% CI 0.68–0.91), with persistent high heterogeneity (I²=93%). There was no evidence of a subgroup difference by study design (*p* = 0.87), although this comparison is limited by the inclusion of only one randomized trial. Across studies with extractable group-level mortality percentages, median mortality was 28.6% (IQR 21.0–33.6%) in ICP-monitored patients and 33.2% (IQR 25.6–42.4%) in non-monitored patients.

Individual study findings remained mixed, with some adjusted analyses favoring ICP monitoring [10, 13, 15, 39, 41, 43, 49, 51] and others showing no benefit or worse outcomes in monitored patients [29, 31, 34, 36, 37, 45, 46, 48]; therefore, the pooled result should be interpreted as a crude association rather than a causal estimate.

**Table 2:** Mortality in ICP-monitored versus non-ICP-monitored groups

Study & Year	ICP monitored					Non-ICP monitored		
	Mortality (*n*)	Total (*n*)	% Mortality	Crude OR [95% CI] of Mortality	Adjusted effect estimate for mortality [95% CI]	Mortality (*n*)	Total (*n*)	% Mortality
Agrawal et al. 2015	118	497	23.7%	0.44 [0.34–0.56]	-	353	848	41.6%
Aiolfi et al. 2017	495	1519	32.6%	1.01 [0.90–1.13]	1.12 [0.983–1.275]	3774	11,669	32.3%
Alali et al. 2013	600	1874	32.0%	0.83 [0.75–0.92]	0.44 [0.31–0.63]	3169	8754	36.2%
Alali et al. 2015	30	273	11.0%	0.71 [0.47–1.06]	0.50 [0.30–0.85]	213	1432	14.9%
Al-Anazi et al. 2004	10	52	19.2%	0.26 [0.12–0.57]	-	60	126	47.6%
Alkhoury et al. 2014	78	285	27.4%	0.84 [0.64–1.10]	0.72 [0.51–1.00]	876	2822	31.0%
Al Saiegh et al. 2020	2134	6025	35.4%	1.34 [1.26–1.42]	aRR 1.03 [0.99–1.08]	8974	30,904	29.0%
Biersteker et al. 2012	59	123	48.0%	1.60 [0.98–2.61]	0.93 [0.47–1.85]	52	142	37%
Chesnut et al. 2012	56	157	35.7%	0.83 [0.53–1.30]	HR 0.91 [0.64–1.30]	67	167	40.1%
Dawes et al. 2015	116	378	30.7%	0.53 [0.39–0.70]	-	203	444	45.7%
Farahvar et al. 2012	212	1084	19.6%	0.49 [0.36–0.67]	0.64 [0.41–1.00]	74	223	33.2%
Foote et al. 2022	20	58	34.5%	0.70 [0.34–1.44]	-	28	65	43.1%
Fu et al. 2020	29	342	8.5%	0.89 [0.52–1.53]	-	29	309	9.4%
Gao et al. 2013	5	39	12.8%	0.74 [0.18–3.06]	-	4	24	16.7%
Griesdale et al. 2010	28	98	28.6%	2.84 [1.25–6.48]	2.76 [1.07–7.08]	9	73	12.3%
Haddad et al. 2011	11	52	21.2%	1.49 [0.73–3.04]	1.71 [0.79–3.70]	65	425	15.3%
Jitchanvichai et al. 2024	8	35	22.9%	0.64 [0.29–1.43]	0.88 [0.27–2.63]	257	814	31.6%
Kostić et al. 2011	15	32	46.9%	0.46 [0.17–1.31]	-	19	29	65.5%
Lane et al. 2000	153	541	28.3%	1.21 [0.99–1.47]	0.77 (*p* = 0.015)	1222	4966	24.6%
Lele et al. 2019	28	126	22.2%	0.42 [0.22–0.78]	aRR 0.50 [0.29–0.87]	30	74	40.5%
Mauritz et al. 2008	402	1031	39.0%	1.04 [0.86–1.26]	-	314	825	38.1%
Nattino et al. 2023	197	503	39.2%	0.94 [0.75–1.17]	-	384	945	40.6%
Peled et al. 2025	194	810	24.0%	0.74 [0.60–0.90]	0.83 [0.65–1.04]	417	1392	30.0%
Robba et al. 2021	441	1318	33.5%	0.52 [0.44–0.61]	HR 0.35 [0.26–0.47]	517	1049	49.3%
Schupper et al. 2019	1257	4093	30.7%	1.19 [1.11–1.28]	-	7236	26,617	27.2%
Shafi et al. 2008	148	708	20.9%	1.95 [1.49–2.55]	1.82 [1.32–2.56]	112	938	11.9%
Suehiro et al. 2017	99	305	32.5%	0.59 [0.44–0.77]	-	354	786	45.0%
Talving et al. 2013	33	101	32.7%	0.42 [0.24–0.72]	0.15 [0.03–0.74]	62	115	53.9%
Yang et al. 2022	139	737	18.9%	0.64 [0.51–0.80]	0.70 [0.54–0.90]	344	1292	26.6%
You et al. 2016	27	80	33.8%	0.49 [0.26–0.91]	-	44	86	51.2%
Zeng et al. 2013	2	77	2.6%	0.22 [0.05–1.02]	-	10	91	11.0%
**Total**	**7144**	**23,353**	**30.6%**	**0.79 [0.68–0.91]**		**29,272**	**98,446**	**29.7%**

NB: Mortality is reported as the number of deaths and percentage within each group. Crude odds ratios (ORs) with 95% confidence intervals (CIs) were calculated from the mortality counts shown when not explicitly reported by the original study. Adjusted effect estimates are presented as reported in the original publication and may therefore be expressed as ORs, hazard ratios (HRs), adjusted relative risks (aRRs), or propensity score–matched/adjusted risk differences where applicable. Where studies reported effects in the reverse direction, these were inverted so that all effect estimates are presented as ICP-monitored versus non-ICP-monitored groups. Values < 1 favour ICP monitoring. Studies without extractable mortality counts or a usable mortality effect estimate were excluded from Table 2

Abbreviations: ICPm, intracranial pressure monitoring; OR, odds ratio; CI, confidence interval; HR, hazard ratio; aRR, adjusted relative risk; PSM, propensity score matching; ICU, intensive care unit; LOS, length of stay; GCS, Glasgow Coma Scale; AIS, Abbreviated Injury Scale; ISS, Injury Severity Score; EVD, external ventricular drain; IP, intraparenchymal; CT, computed tomography

### Duration of stay

Length-of-stay outcomes were reported in 20 studies [14, 15, 29, 31, 32, 34–39, 43–47, 49, 50, 53, 55] and are summarized in Table 3. ICU length of stay was reported in 18 studies [14, 15, 29, 31, 32, 34, 36–39, 43–45, 47, 49, 50, 53, 55], while hospital length of stay was reported in 16 studies [14, 15, 29, 32, 35, 37–39, 43–47, 50, 53, 55]. Across most studies with extractable data, ICP-monitored patients had longer ICU and hospital stays than non-monitored patients. ICU LOS in monitored patients ranged from approximately 6 to 18 days, although reporting formats varied between studies and included means, medians, interquartile ranges, standard deviations, standard errors, and mean-only values. Hospital LOS was similarly heterogeneous, ranging from approximately 12 to 87 days in monitored patients. Heterogeneity of LOS between studies lacked further interpretive value.

**Table 3:** Length of stay in ICP-monitored versus non-ICP-monitored groups

Authors & Year	ICU LOS (days)		Hospital LOS (days)	
	ICPm	No ICPm	ICPm	No ICPm
Aiolfi et al. 2017	14 (9–21)	5 (3–11)	20 (13–30)	9 (4–17)
Al Saiegh et al. 2020	13.1 ± 11.6	6.0 ± 10.8	18.97 ± 18.71	9.68 ± 14.74
Alkhoury et al. 2014	12.6 ± 10.3	6.3 ± 8.7	21.0 ± 19.5	10.4 ± 14.9
Biersteker et al. 2012	10.8 (4.2–21)	2.65 (1.00–6.9)	22.0 (8.3–44)	7.48 (1.9–20)
Chesnut et al. 2012	12 (6–17)	9 (6–16)	NR	NR
Foote et al. 2022	11 (3–19)	3 (1–9)	12 (5–25)	3 (2–11)
Fu et al. 2020	NR	NR	20 (15–33)	21 (16–30)
Gao et al. 2013	15.67 ± 8.72	25.32 ± 18.78	18.94 ± 8.92	34.29 ± 22.64
Griesdale et al. 2010	14 (11)	6 (8)	NR	NR
Haddad et al. 2011	17.0 ± 8.8	11.6 ± 8.3	86.7 ± 81.9	78.4 ± 332.2
Lane et al. 2000	9.7 (mean only)	4.3 (mean only)	44.0 (mean only)	22.8 (mean only)
Lele et al. 2019	6.1 ± 1.8	5.9 ± 2.5	NR	NR
Mauritz et al. 2008	18 (8–31)	9 (5–17)	NR	NR
Nattino et al. 2023	18.0 (12.0–26.0)	12.0 (5.0–20.0)	27.0 (17.8–39.0)	20.0 (10.0–34.0)
Peled et al. 2025	15 (7–26)	5 (2–15)	29 (14–42)	10 (4–26)
Schupper et al. 2019	11 (6–17)	4 (2–10)	15 (8–26)	12 (6–21)
Shafi et al. 2008	NR	NR	25 ± 0.8	22 ± 0.7
Talving et al. 2013	16.8 ± 1.3	8.2 ± 1.0	19.4 ± 1.6	10.1 ± 1.2
Yang et al. 2022	11.79	7.95	25.96	21.71
You et al. 2016	14.3 ± 6.4	11.6 ± 5.8	28.5 ± 12.1	26.1 ± 13.5

NB: Length-of-stay data are reported as mean ± SD, mean ± SEM, median (IQR), median (range), or mean only, as presented in the original publication. Units are days unless otherwise specified. “NR” indicates that no extractable comparable ICU or hospital LOS value was reported

Abbreviations: ICPm, intracranial pressure monitoring; ICU, intensive care unit; LOS, length of stay; IQR, interquartile range; SD, standard deviation; SEM, standard error of the mean; NR, not reported

### Complications

Ten studies (32%) reported extractable complication data by ICP-monitored versus non-ICP-monitored groups or device-specific complications within monitored patients [13, 14, 29, 31, 33, 35, 40, 43, 46, 47]. The most frequently reported complications were infections, hemorrhagic events, and mechanical device failures.

Three studies (10%) reported ICP monitor–related infection specifically, with reported rates of 2.0% to 3.8% among monitored patients [13, 43, 46]. Broader infection-related outcomes, including pneumonia, sepsis, and composite infection categories, were reported in eight studies (26%) [13, 14, 29, 31, 35, 43, 46, 47]. Hemorrhagic complications related to ICP monitor insertion were reported in two studies (6.5%) with reported rates of 1.0% to 8.7% [31, 43]. Mechanical device-related complications were reported heterogeneously in three studies (10%) [13, 31, 33].

Detailed complication data are provided in Supplemental Digital Content 6 g (SDC 6 g).

### Functional outcomes

Ten studies (32%) reported GOS or GOS-E/GOSE outcomes [13, 14, 31, 33, 38, 40, 43, 46, 49, 50]. Nine (29%) studies reported extractable group-level GOS/GOSE comparisons between ICP-monitored and non-monitored patients [14, 31, 33, 38, 40, 43, 46, 49, 50], while Robba et al. reported adjusted GOSE < 5 subgroup analyses only [13]. Four (13%) studies reported better GOS/GOSE outcomes with ICPm [33, 40, 43, 50], two (6.5%) showed worse GOS/GOSE outcomes in monitored patients [14, 38], and three (10%) showed no clear difference after adjustment, randomization, or propensity matching [31, 46, 49]. Two studies (6.5%) reported group-level FIM scores, both showing worse functional independence in ICP-monitored patients [35, 45]. Aiolfi et al. additionally reported lower good functional independence among monitored survivors [29]. Detailed functional outcome data are provided in Supplemental Digital Content 6b–f (SDC 6b–f).

## Discussion

Although ICPm and ICP-directed therapy are well-established practices in severe TBI, their impact on functional neurological recovery and survival remains uncertain.

### Mortality

Mortality findings were inconsistent across studies. Several previous systematic reviews reported lower mortality with ICPm. Yuan et al. found a significant reduction in mortality in patients undergoing ICPm (adjusted OR 0.71, 95% CI 0.62–0.82, *p* < 0.001) [12]. Likewise, this meta-analysis of crude mortality data showed lower mortality with ICPm, but substantial heterogeneity, reflecting clinical and methodological variability between included studies, limits causal interpretation. Several observational studies reported lower adjusted or crude mortality with ICPm, including Robba et al., Alali et al., Talving et al., Kostić et al., and Mauritz et al.; however, these findings were accompanied by longer ICU or hospital stays and did not consistently translate into improved functional outcomes [13, 30, 34, 39, 41]. 

In contrast, the randomized trial by Chesnut et al. found no significant mortality benefit with ICP-guided care at 6 months (39% vs. 41%; HR 0.91, 95% CI 0.64–1.30) [31]. Shafi et al. also reported worse adjusted survival among ICP-monitored patients [35], while Griesdale et al. found higher adjusted hospital mortality in patients managed with external ventricular drains [36]. 

The optimal timing of ICP monitor insertion remains uncertain and may confound outcome interpretation, because early monitor placement may reflect either timely neurocritical care or greater early injury severity. Timing-focused studies were not included in the primary analysis when they did not compare monitored versus unmonitored patients, but they remain relevant to interpretation. A recent systematic review found no clear mortality difference between early and late ICP monitoring, while protocolization studies have mainly assessed monitor utilization and timeliness rather than the independent effect of ICPm on mortality or functional outcomes [56, 57]. 

The apparent effect of ICPm may also be modified by treatment era. Earlier studies were conducted before widespread adoption of modern guideline-based neurocritical care, when invasive ICP data may have provided a larger incremental benefit. In more recent cohorts, improved imaging, resuscitation, ICU protocols, decompressive surgery, and multimodal care may reduce the marginal detectable effect of ICPm, particularly when monitored and unmonitored groups differ substantially in baseline severity [16, 58, 59]. 

Age and injury severity may modify the effect of ICPm. Several registry studies suggest that older patients are less likely to receive monitoring and may derive less apparent benefit, whereas younger patients and those with severe physiological risk but potentially salvageable injury may represent the population most likely to benefit [13, 44, 45, 55]. However, few studies provided sufficiently granular subgroup data to support reliable pooled subgroup analysis.

### Functional outcomes

Functional outcomes following ICPm were heterogeneous and inconsistently reported. Robba et al. reported improved 6-month neurological outcomes with ICPm in patients with at least one unreactive pupil, but not in less severe subgroups [13]. Several studies reported better GOS/GOS-E outcomes in monitored patients, including Al-Anazi et al., You et al., Zeng et al., and Gao et al. [33, 40, 43, 50]; however, other studies found no clear functional benefit after randomization, adjustment, or propensity matching [31, 46, 49]. In contrast, Biersteker et al. and Nattino et al. reported worse GOS/GOS-E outcomes in monitored patients [14, 38], while Shafi et al., Aiolfi et al., and Al Saiegh et al. reported worse functional independence among monitored patients [29, 35, 45]. Overall, the available evidence did not demonstrate a consistent functional outcome benefit associated with ICPm.

The lack of consistent functional outcome benefit is important when considering the long-term cost-effectiveness of ICPm and its impact on patients, families, and health systems. This aspect requires careful assessment to balance the goal of survival against the potential for meaningful functional recovery.

### Length of stay and resource utilization

Most studies reporting length-of-stay outcomes found longer ICU or hospital stays in ICP-monitored patients. This likely reflects both greater treatment intensity and confounding by injury severity, but also indicates higher short-term resource utilization among monitored patients [14, 15, 29, 31, 32, 34–39, 43–47, 49, 50, 53, 55]. 

### Complications and risks

Insertion of ICP monitors is invasive and associated with device-related and systemic complications. ICP monitor–related infection was reported in three studies, with rates of 2.0–3.8% among monitored patients [13, 43, 46]. Broader infection-related complications, including pneumonia, sepsis, and composite infection categories, were reported in eight studies [13, 14, 29, 31, 35, 43, 46, 47]. Mechanical device-related complications were reported in three studies: Chesnut et al. reported catheter malfunction and unplanned catheter removal 3%; Al-Anazi et al. reported catheter obstruction in 3.8% and Robba et al. reported catheter misposition in 13%, accidental catheter removal in 9% and faulty or broken catheters in 11% [13, 31, 33]. Hemorrhagic complications related to ICP monitoring were reported in two studies: Chesnut et al. reported catheter-related hemorrhage in 1%, while You et al. reported hemorrhage in 8.7% [31, 43]. Fu et al. reported lower rates of hydrocephalus and CSF leak in monitored patients compared with unmonitored patients (0.9% vs. 3.2%, *p* = 0.026; and 0.6% vs. 3.6%, *p* = 0.004, respectively) [46]. Overall, complication reporting was heterogeneous, and procedural factors such as operator experience, insertion technique, and monitor duration were poorly reported.

### Variability in ICP monitoring practices

ICPm practice varied substantially across regions and health systems. Included studies were conducted in high-resource, middle-income, and resource-limited settings, including North America, Europe, Asia, the Middle East, South America, and one international cohort across 42 countries [10, 13–15, 29–55]. Older patients were less likely to receive ICPm in several registry studies, including Schupper et al., Al Saiegh et al., and Peled et al. [44, 45, 55] Institutional factors also influenced ICPm use; Alali et al. reported lower mortality in hospitals with higher ICPm utilization, while Aiolfi et al. reported variable compliance with Brain Trauma Foundation ICPm criteria across trauma centers [29, 41]. These findings support the need for clearer, generalizable ICPm selection criteria that account for injury severity, age, available neurosurgical resources, and local health-system context.

### Limitations

The lack of consistent reporting standards across studies limited comparison across outcomes and restricted meta-analysis to mortality. Functional outcomes were particularly heterogeneous. Ten studies reported GOS or GOS-E/GOSE outcomes, although only nine provided extractable group-level comparisons between ICP-monitored and non-monitored patients, while Robba et al. reported adjusted GOSE < 5 subgroup analyses only [13, 14, 31, 33, 38, 40, 43, 46, 49, 50]. Two studies reported group-level FIM scores, both at discharge, and Aiolfi et al. reported functional independence categories among survivors [29, 35, 45]. Other studies reported discharge disposition or dichotomized favorable/unfavorable outcomes, limiting direct comparison across studies. This heterogeneity in outcome measure, timing, and reporting format limited interpretability of functional recovery after ICPm.

The primary limitation of this review is that most included studies were observational, with only one randomized controlled trial [31]. As a result, mortality and functional outcome findings remain vulnerable to confounding by indication, because patients selected for ICPm often differed systematically from non-monitored patients in baseline neurological severity, CT findings, systemic injury burden, and treatment intensity. Approaches to adjustment were heterogeneous across studies. Age and GCS were the most commonly adjusted variables, whereas pupillary response, CT-defined injury severity, extracranial injury burden, and physiological severity were less consistently incorporated. Several studies used multivariable regression, propensity score matching, inverse probability weighting, or multilevel modeling, but residual confounding remained likely because monitor placement was clinician-directed and key variables related to severity of neurological injury were often incompletely reported [10, 13–15, 29, 32, 34, 38, 39, 41, 42, 44–46, 48, 49, 51]. 

A further limitation is that the mortality meta-analysis pooled crude odds ratios derived from extractable group-level mortality counts. Adjusted estimates were not pooled because they were inconsistently reported and varied in effect measure, model specification, covariate selection, and timing of outcome assessment. Therefore, the pooled mortality estimate should be interpreted as a crude association rather than a causal estimate of the effect of ICPm.

Further, large registry studies contributed substantial statistical weight to the pooled crude analysis, but these datasets may have limited physiological granularity and may not adequately capture clinician selection, timing of monitor insertion, CT severity, or center-level expertise. The inclusion of pediatric and mixed-age cohorts, despite the adult-only criterion in the registered protocol may contribute to clinical heterogeneity.

### Implications for practice and research

The variability in outcome reporting and incomplete adjustment for clinically important confounders highlight the need for consensus on study design, core outcome measures, and minimum adjustment variables in ICPm research. Randomized trials are difficult to conduct in this context and may not be feasible or ethical in many health systems; however, well-designed prospective cohorts, registry-based comparative effectiveness studies, and natural experiments comparing centers with different ICPm utilization rates remain feasible [31, 41]. Future studies should define which patients are most likely to benefit from ICPm, and should examine the optimal timing, duration, and modality of monitoring [8, 13, 56, 57]. Cost-effectiveness analyses are also needed, particularly in resource-limited settings, where the opportunity cost of invasive monitoring, prolonged ICU stay, and downstream rehabilitation burden may be substantial. Research into less invasive or lower-risk monitoring strategies may help reduce procedural complications and broaden access to physiologically guided neurocritical care.

Future research should include prospective comparative-effectiveness designs and carefully developed trials where feasible. The Benchmark Evidence from Latin America–Treatment of Raised Intracranial Pressure–Pediatrics (BELA-TRIPP) trial-development work demonstrates one approach to designing an ethically acceptable ICP-monitoring trial in a severely head injured pediatric cohort using imaging and examination-based management as the comparator [60, 61]. 

The interpretation of ICPm literature is limited by the tension between physiological plausibility, historical practice, and observational confounding. Raised ICP is biologically harmful and ICPm provides actionable physiological information, yet comparative studies often evaluate monitoring in populations where monitor placement is strongly influenced by clinician judgment, injury severity, age, center expertise, and treatment era. Large registry studies provide statistical power but frequently lack detailed physiological, imaging, treatment-threshold, and timing data. Conversely, smaller prospective cohorts may provide richer clinical detail but contribute less weight to pooled estimates. These factors mean that crude meta-analysis may obscure benefit in selected subgroups while over-representing large administrative datasets with limited granularity. Accordingly, the present meta-analysis should not be interpreted as a definitive causal estimate of ICPm effectiveness. Rather, it demonstrates that the comparative literature remains heterogeneous, incompletely adjusted, and inconsistent with respect to both mortality and functional recovery. Future work should prioritize prospective comparative-effectiveness designs, center-level natural experiments, and subgroup analyses focused on age, pupillary response, CT phenotypes, timing of insertion, monitor modality, and treatment intensity.

## Conclusion

This systematic review found a crude association between ICPm and lower mortality in severe TBI; however, substantial heterogeneity and residual confounding limit causal interpretation. Functional neurological outcomes were inconsistently reported and did not show a consistent benefit with ICPm. Future research should prioritize standardized functional outcome reporting, clearer adjustment for key confounders, and prospective comparative designs to define which patients are most likely to benefit from ICPm.

## Supplementary Information

Below is the link to the electronic supplementary material.

Supplementary Material 1

Supplementary Material 2

Supplementary Material 3

Supplementary Material 4

Supplementary Material 5

Supplementary Material 6

## Data Availability

Data extracted and analyzed for this review are available from the corresponding author upon reasonable request.

## References

[1] Salmond CH, Sahakian BJ. Cognitive outcome in traumatic brain injury survivors. Curr Opin Crit Care. 2005;11:111–6. 10.1097/01.ccx.0000155358.31983.37. 15758589 10.1097/01.ccx.0000155358.31983.37

[2] Arciniegas DB, Held K, Wagner P. Cognitive impairment following traumatic brain injury. Curr Treat Options Neurol [Internet]. 2002 [cited 2026 May 9];4:43–57. 10.1007/s11940-002-0004-6. 10.1007/s11940-002-0004-6 11734103

[3] Shuanglong Z, Jiangyuan Y, Meng N, Zheng W, Yunshui Z, Wei S, et al. A meta-analysis of cognitive and functional outcomes in severe brain trauma cases. Front Behav Neurosci [Internet]. Frontiers; 2024 [cited 2026 May 9];18. 10.3389/fnbeh.2024.1349672. 10.3389/fnbeh.2024.1349672 PMC10972858 38549619

[4] Crozes F, Delpierre C, Costa N. Mapping the costs and socioeconomic characteristics involved in, traumatic brain injuries: a scoping review. J Rehabil Med [Internet]. 2024 [cited 2026 May 9];56:18311. 10.2340/jrm.v56.18311. 39101675 10.2340/jrm.v56.18311 PMC11318505

[5] National Academies of Sciences E, Division H and M, Services B on HC, Policy B on HS, Care C on AP in TBIR and, Matney C, et al. The Scope and Burden of Traumatic Brain Injury. Traumatic Brain Injury: A Roadmap for Accelerating Progress [Internet]. National Academies Press (US); 2022 [cited 2026 May 9]. https://www.ncbi.nlm.nih.gov/books/NBK580076/. Accessed 9 May 2026. 35533242

[6] James S, Theadom A, Ellenbogen R, Bannick M, Mountjoy-Venning WC, Lucchesi L. Global, regional, and national burden of traumatic brain injury and spinal cord injury, 1990–2016: a systematic analysis for the Global Burden of Disease Study 2016. Lancet Neurol. 2019;18:56–87. 10.1136/bmjopen-2023-075049. 30497965 10.1016/S1474-4422(18)30415-0 PMC6291456

[7] Liu M-W, Ma Z-Q, Liao R-L, Chen W-M, Zhang B-R, Zhang Q-J, et al. Incidence and mortality related risk factors in patients with severe traumatic brain injury: A meta–analysis. Exp Ther Med [Internet]. 2025 [cited 2025 July 13];29:84. 10.3892/etm.2025.12834. 10.3892/etm.2025.12834 PMC11904872 40084190

[8] Florez-Perdomo WA, Mishra R, Moscote-Salar LR, Cincu R, Maurya VP, Agrawal A. Intracranial pressure monitoring in patients with traumatic brain injury: an umbrella review of systematic review and meta-analysis. J Neurointensive Care. 2024;7:18–28. 10.32587/jnic.2023.00731.

[9] Majdan M, Plancikova D, Maas A, Polinder S, Feigin V, Theadom A, et al. Years of life lost due to traumatic brain injury in Europe: A cross-sectional analysis of 16 countries. PLoS Med. 2017;14:e1002331. 10.1371/journal.pmed.1002331. 28700588 10.1371/journal.pmed.1002331 PMC5507416

[10] Farahvar A, Gerber LM, Chiu Y-L, Carney N, Härtl R, Ghajar J. Increased mortality in patients with severe traumatic brain injury treated without intracranial pressure monitoring: Clinical article. JNS [Internet]. 2012 [cited 2026 Apr 4];117:729–34. 10.3171/2012.7.JNS111816. 10.3171/2012.7.JNS111816 22900846

[11] de Macedo Filho L, Figueredo LF, Villegas-Gomez GA, Arthur M, Pedraza-Ciro MC, Martins H, et al. Pathophysiology-Based Management of Secondary Injuries and Insults in TBI. Biomedicines [Internet]. Multidisciplinary Digital Publishing Institute; 2024 [cited 2025 July 13];12:520. 10.3390/biomedicines12030520. 10.3390/biomedicines12030520 PMC10968249 38540133

[12] Yuan Q, Wu X, Sun Y, Yu J, Li Z, Du Z, et al. Impact of intracranial pressure monitoring on mortality in patients with traumatic brain injury: a systematic review and meta-analysis. Journal of Neurosurgery [Internet]. American Association of Neurological Surgeons; 2015 [cited 2025 Aug 7];122:574–87. 10.3171/2014.10.JNS1460. 10.3171/2014.10.JNS1460 25479125

[13] Robba C, Graziano F, Rebora P, Elli F, Giussani C, Oddo M, et al. Intracranial pressure monitoring in patients with acute brain injury in the intensive care unit (SYNAPSE-ICU): an international, prospective observational cohort study. Lancet Neurol [Internet]. 2021 [cited 2026 Apr 4];20:548–58. 10.1016/S1474-4422(21)00138-1. 10.1016/S1474-4422(21)00138-1 34146513

[14] Nattino G, Gamberini L, Brissy O, Carrara G, Chesnut R, Chiarini V, et al. Comparative Effectiveness of Intracranial Pressure Monitoring on 6-Month Outcomes of Critically Ill Patients With Traumatic Brain Injury. JAMA Netw Open [Internet]. 2023 [cited 2026 Apr 4];6:e2334214. 10.1001/jamanetworkopen.2023.34214. 37755832 10.1001/jamanetworkopen.2023.34214 PMC10534270

[15] Yang C, Ma Y, Xie L, Wu X, Hui J, Jiang J, et al. Intracranial Pressure Monitoring in the Intensive Care Unit for Patients with Severe Traumatic Brain Injury: Analysis of the CENTER-TBI China Registry. Neurocrit Care [Internet]. 2022 [cited 2026 Apr 4];37:160–71. 10.1007/s12028-022-01463-w. 10.1007/s12028-022-01463-w 35246788

[16] Carney N, Totten AM, O’Reilly C, Ullman JS, Hawryluk GWJ, Bell MJ, et al. Guidelines for the Management of Severe Traumatic Brain Injury, Fourth Edition. Neurosurgery [Internet]. 2017 [cited 2023 Mar 19];80:6–15. 10.1227/NEU.0000000000001432. 10.1227/NEU.0000000000001432 27654000

[17] Brain Trauma Foundation, American Association of Neurological Surgeons, Congress of Neurological Surgeons, Joint Section on Neurotrauma and Critical, Care AANSCNS, Bratton SL, Chestnut RM, et al. Guidelines for the management of severe traumatic brain injury. VI. Indications for intracranial pressure monitoring. J Neurotrauma. 2007;24 Suppl 1:S37-44. 10.1089/neu.2007.9990. 10.1089/neu.2007.9990 17511544

[18] Page MJ, McKenzie JE, Bossuyt PM, Boutron I, Hoffmann TC, Mulrow CD. The PRISMA 2020 statement: an updated guideline for reporting systematic reviews. BMJ. 2021;372:n71. 33782057 10.1136/bmj.n71 PMC8005924

[19] Kim N, Balogh Z. CRD42025643607: Impacts of intracranial pressure monitoring on mortality and length of stay in patients with severe traumatic brain injury: A systematic review. PROSPERO 2024 [Internet]. 2025 [cited 2026 May 9]. https://www.crd.york.ac.uk/PROSPERO/view/CRD42025643607. Accessed 9 May 2026.

[20] Velli G, Bright M, Del Vecchio L. Enhancing Systematic Reviews in Health Science Using Integrated Search Interfaces, Discovery Layers and Federated Searching. J Hosp Librariansh [Internet]. 2024 [cited 2026 Jan 7];24:185–95. 10.1080/15323269.2024.2365088.

[21] Hirt J, Nordhausen T, Fuerst T, Ewald H, Appenzeller-Herzog C. TARCiS study group. Guidance on terminology, application, and reporting of citation searching: the TARCiS statement. BMJ. 2024;385:e078384. 10.1136/bmj-2023-078384. 38724089 10.1136/bmj-2023-078384

[22] The EndNote Team. EndNote. EndNote 2025. Philadelphia, PA: Clarivate; 2013.

[23] Microsoft Corporation. Microsoft Excel for Mac. 2022.

[24] von Elm E, Altman DG, Egger M, Pocock SJ, Gøtzsche PC, Vandenbroucke JP, et al. The Strengthening the Reporting of Observational Studies in Epidemiology (STROBE) statement: guidelines for reporting observational studies. J Clin Epidemiol. 2008;61:344–9. 10.1016/j.jclinepi.2007.11.008. 18313558 10.1016/j.jclinepi.2007.11.008

[25] Sterne JAC, Savović J, Page MJ, Elbers RG, Blencowe NS, Boutron I, et al. RoB 2: a revised tool for assessing risk of bias in randomised trials. BMJ [Internet]. BMJ Group; 2019 [cited 2026 Jan 7];366:l4898. 10.1136/bmj.l4898. 10.1136/bmj.l4898 31462531

[26] Campbell M, McKenzie JE, Sowden A, Katikireddi SV, Brennan SE, Ellis S, et al. Synthesis without meta-analysis (SWiM) in systematic reviews: reporting guideline. BMJ [Internet]. BMJ Group; 2020 [cited 2026 Jan 7];368:l6890. 10.1136/bmj.l6890. 10.1136/bmj.l6890 PMC7190266 31948937

[27] Jeremy H, Iain C, Paul G, Trish G, Carl H, Alessandro L. Explanation of the 2011 Oxford Centre for Evidence-Based Medicine (OCEBM) Levels of Evidence Background Document [Internet]. 2011. https://www.cebm.ox.ac.uk/resources/levels-of-evidence/ocebm-levels-of-evidence.

[28] Aiolfi A, Benjamin E, Khor D, Inaba K, Lam L, Demetriades D. Erratum to: Brain Trauma Foundation Guidelines for Intracranial Pressure Monitoring: Compliance and Effect on Outcome. World J Surg. 2017;41:1542. 10.1007/s00268-017-3913-y. 28224197 10.1007/s00268-017-3913-y

[29] Aiolfi A, Benjamin E, Khor D, Inaba K, Lam L, Demetriades D. Brain Trauma Foundation Guidelines for Intracranial Pressure Monitoring: Compliance and Effect on Outcome. World J Surg [Internet]. 2017 [cited 2026 Apr 4];41:1543–9. 10.1007/s00268-017-3898-6. 10.1007/s00268-017-3898-6 28188356

[30] Kostic A, Stefanovic I, Novak V, Veselinovic D, Ivanov G, Veselinovic A. Prognostic significance of intracranial pressure monitoring and intracranial hypertension in severe brain trauma patients. Med pregl [Internet]. 2011 [cited 2026 Apr 4];64:461–5. 10.2298/MPNS1110461K. 22097111

[31] Chesnut RM, Temkin N, Carney N, Dikmen S, Rondina C, Videtta W, et al. A Trial of Intracranial-Pressure Monitoring in Traumatic Brain Injury. N Engl J Med [Internet]. Massachusetts Medical Society; 2012 [cited 2026 Apr 4];367:2471–81. 10.1056/NEJMoa1207363. 10.1056/NEJMoa1207363 PMC3565432 23234472

[32] Lane PL, Skoretz TG, Doig G, Girotti MJ. Intracranial pressure monitoring and outcomes after traumatic brain injury. Can J Surg. 2000;43:442–8. 11129833 PMC3695200

[33] Al-Anazi AH. Experience with severe head injury and role of intracranial pressure monitoring in Eastern Saudi Arabia. Neurosciences (Riyadh). Riyadh Saudi Arabia. 2004;9:265–70. 23377246

[34] Mauritz W, Steltzer H, Bauer P, Dolanski-Aghamanoukjan L, Metnitz P. Monitoring of intracranial pressure in patients with severe traumatic brain injury: an Austrian prospective multicenter study. Intensive Care Med [Internet]. 2008 [cited 2026 Apr 4];34:1208–15. 10.1007/s00134-008-1079-7. 18365169 10.1007/s00134-008-1079-7

[35] Shafi S, Diaz-Arrastia R, Madden C, Gentilello L. Intracranial Pressure Monitoring in Brain-Injured Patients is Associated With Worsening of Survival. J Trauma [Internet]. 2008 [cited 2026 Apr 4];64:335–40. 10.1097/TA.0b013e31815dd017. 18301195 10.1097/TA.0b013e31815dd017

[36] Griesdale DEG, McEwen J, Kurth T, Chittock DR. External Ventricular Drains and Mortality in Patients with Severe Traumatic Brain Injury. Can J Neurol Sci [Internet]. 2010 [cited 2026 Apr 4];37:43–8. 10.1017/S031716710000963X. 10.1017/s031716710000963x 20169772

[37] Haddad S, Aldawood AS, Alferayan A, Russell NA, Tamim HM, Arabi YM. Relationship between Intracranial Pressure Monitoring and Outcomes in Severe Traumatic Brain Injury Patients. Anaesth Intensive Care [Internet]. 2011 [cited 2026 Apr 4];39:1043–50. 10.1177/0310057X1103900610. 22165356 10.1177/0310057X1103900610

[38] Biersteker HAR, Andriessen TMJC, Horn J, Franschman G, Van Der Naalt J, Hoedemaekers CWE et al. Factors influencing intracranial pressure monitoring guideline compliance and outcome after severe traumatic brain injury*: Critical Care Medicine [Internet]. 2012 [cited 2026 Apr 4];40:1914–22. 10.1097/CCM.0b013e3182474bde. 10.1097/CCM.0b013e3182474bde 22488001

[39] Talving P, Karamanos E, Teixeira PG, Skiada D, Lam L, Belzberg H et al. Intracranial pressure monitoring in severe head injury: compliance with Brain Trauma Foundation guidelines and effect on outcomes: a prospective study: Clinical article. JNS [Internet]. 2013 [cited 2026 Apr 4];119:1248–54. 10.3171/2013.7.JNS122255. 10.3171/2013.7.JNS122255 23971954

[40] Zeng J, Tong W, Zheng P. Decreased risk of acute kidney injury with intracranial pressure monitoring in patients with moderate or severe brain injury: Clinical article. JNS [Internet]. 2013 [cited 2026 Apr 4];119:1228–32. 10.3171/2013.7.JNS122131. 10.3171/2013.7.JNS122131 23909252

[41] Alali AS, Fowler RA, Mainprize TG, Scales DC, Kiss A, De Mestral C, et al. Intracranial Pressure Monitoring in Severe Traumatic Brain Injury: Results from the American College of Surgeons Trauma Quality Improvement Program. J Neurotrauma [Internet]. 2013 [cited 2026 Apr 4];30:1737–46. 10.1089/neu.2012.2802. 23731257 10.1089/neu.2012.2802 PMC3796332

[42] Dawes AJ, Sacks GD, Cryer HG, Gruen JP, Preston C, Gorospe D, et al. Intracranial pressure monitoring and inpatient mortality in severe traumatic brain injury: A propensity score–matched analysis. J Trauma Acute Care Surg [Internet]. 2015 [cited 2026 Apr 4];78:492–502. 10.1097/TA.0000000000000559. 25710418 10.1097/TA.0000000000000559

[43] You W, Feng J, Tang Q, Cao J, Wang L, Lei J, et al. Intraventricular intracranial pressure monitoring improves the outcome of older adults with severe traumatic brain injury: an observational, prospective study. BMC Anesthesiol [Internet]. 2015 [cited 2026 Apr 4];16:35. 10.1186/s12871-016-0199-9. 10.1186/s12871-016-0199-9 PMC4940906 27401211

[44] Schupper AJ, Berndtson AE, Smith A, Godat L, Costantini TW. Respect your elders: effects of ageing on intracranial pressure monitor use in traumatic brain injury. Trauma Surg Acute Care Open [Internet]. 2019 [cited 2026 Apr 4];4:e000306.10.1136/tsaco-2019-000306. 31321312 10.1136/tsaco-2019-000306 PMC6598557

[45] Al Saiegh F, Philipp L, Mouchtouris N, Chalouhi N, Khanna O, Shah SO, et al. Comparison of Outcomes of Severe Traumatic Brain Injury in 36,929 Patients Treated with or without Intracranial Pressure Monitoring in a Mature Trauma System. World Neurosurgery [Internet]. 2020 [cited 2026 Apr 4];136:e535–41. 10.1016/j.wneu.2020.01.070. 10.1016/j.wneu.2020.01.070 31954892

[46] Fu P, Yuan Q, Lv K, Hu J. First Intracranial Pressure Monitoring or First Operation: Which One Is Better? World Neurosurgery [Internet]. 2020 [cited 2026 Apr 4];133:e105–14. 10.1016/j.wneu.2019.08.166. 10.1016/j.wneu.2019.08.166 31479786

[47] Foote CW, Jarvis S, Doan X-L, Guice J, Cruz B, Vanier C, et al. Correlation between intracranial pressure monitoring for severe traumatic brain injury with hospital length of stay and discharge disposition: a retrospective observational cohort study. Patient Saf Surg [Internet]. 2022 [cited 2026 Apr 4];16:40. 10.1186/s13037-022-00350-9. 36581936 10.1186/s13037-022-00350-9 PMC9801642

[48] Jitchanvichai J, Tunthanathip T. Effect of intracranial pressure monitoring on mortality following severe traumatic brain injury in Thailand: propensity score matching methods. J Emerg Crit Care Med [Internet]. 2024 [cited 2026 Apr 4];8:1–1. 10.21037/jeccm-23-109.

[49] Lele A, Kannan N, Vavilala MS, Sharma D, Mossa-Basha M, Agyem K, et al. Patients Who Benefit from Intracranial Pressure Monitoring without Cerebrospinal Fluid Drainage After Severe Traumatic Brain Injury. Neurosurg [Internet]. 2019 [cited 2026 Apr 4];85:231–9. 10.1093/neuros/nyy247. 10.1093/neuros/nyy247 30053135

[50] Gao L, Wu X, Hu J, Jin Y, Han X, Wu X, et al. Intensive management and prognosis of 127 cases with traumatic bilateral frontal contusions. World Neurosurg United States. 2013;80:879–88. 10.1016/j.wneu.2013.01.020. 10.1016/j.wneu.2013.01.020 23313235

[51] Agrawal D, Raghavendran K, Schaubel DE, Mishra MC, Rajajee V. A Propensity Score Analysis of the Impact of Invasive Intracranial Pressure Monitoring on Outcomes after Severe Traumatic Brain Injury. J Neurotrauma [Internet]. SAGE Publications; 2016 [cited 2026 Apr 4];33:853–8. 10.1089/neu.2015.4015. 10.1089/neu.2015.4015 26414629

[52] Alali AS, Gomez D, Sathya C, Burd RS, Mainprize TG, Moulton R, et al. Intracranial pressure monitoring among children with severe traumatic brain injury. PED [Internet]. 2015 [cited 2026 Apr 4];16:523–32. 10.3171/2015.3.PEDS14507. 10.3171/2015.3.PEDS14507 26273741

[53] Alkhoury F, Kyriakides TC. Intracranial Pressure Monitoring in Children With Severe Traumatic Brain Injury: National Trauma Data Bank–Based Review of Outcomes. JAMA Surg [Internet]. 2014 [cited 2026 Apr 4];149:544. 10.1001/jamasurg.2013.4329. 10.1001/jamasurg.2013.4329 24789426

[54] Suehiro E, Fujiyama Y, Koizumi H, Suzuki M, The Japan Neurotrauma Data Bank Com. Directions for Use of Intracranial Pressure Monitoring in the Treatment of Severe Traumatic Brain Injury Using Data from The Japan Neurotrauma Data Bank. J Neurotrauma [Internet]. 2017 [cited 2026 Apr 4];34:2230–4. 10.1089/neu.2016.4948. 10.1089/neu.2016.4948 28335668

[55] Peled A, Kimchi G, Givon A, Gardner RC, Knoller N, Katorza E, et al. The impact of intracranial pressure monitoring in severe traumatic brain injury: results from the National Trauma Registry. Journal of Neurosurgery [Internet]. 2025 [cited 2026 Apr 4];142:1625–34. 10.3171/2024.10.JNS24502. 39951697 10.3171/2024.10.JNS24502

[56] Abdollahifard S, Moshfeghinia R, Najibi A, Moradi M, Motiei-Langroudi R. Timing of intracranial pressure (ICP) monitoring in traumatic brain injury (TBI): a systematic review and meta-analysis. World Neurosurg. 2025. 10.1016/j.wneu.2025.124136. 40449835 10.1016/j.wneu.2025.124136

[57] Beach LK, Todor RD, Petrone P, Liveris A, Reddy S, Torres-Acevedo N. The impact of a protocolization approach to increase the use of and timeliness to intracranial pressure monitoring in patients with severe traumatic brain injury at a level 1 trauma center. Am Surg. 2025;91:911–4. 10.1177/00031348251318392. 39894784 10.1177/00031348251318392

[58] Stein SC, Georgoff P, Meghan S, Mizra K, Sonnad SS. 150 Years of Treating Severe Traumatic Brain Injury: A Systematic Review of Progress in Mortality. Journal of Neurotrauma [Internet]. SAGE Publications; 2010 [cited 2026 May 9];27:1343–53. 10.1089/neu.2009.1206. 10.1089/neu.2009.1206 20392140

[59] Shen L, Wang Z, Su Z, Qiu S, Xu J, Zhou Y, et al. Effects of Intracranial Pressure Monitoring on Mortality in Patients with Severe Traumatic Brain Injury: A Meta-Analysis. PLOS ONE [Internet]. Public Library of Science; 2016 [cited 2026 May 9];11:e0168901. 10.1371/journal.pone.0168901. 10.1371/journal.pone.0168901 PMC5193438 28030638

[60] Chesnut R, Temkin N, Pridgeon J, Sulzbacher S, Lujan S, Videtta W, et al. Development of a Randomized Trial Comparing ICP-Monitor–Based Management of Severe Pediatric Traumatic Brain Injury to Management Based on Imaging and Clinical Examination Without ICP Monitoring–Study Protocol. Neurosurgery [Internet]. 2024 [cited 2026 May 9];94:65–71. 10.1227/neu.0000000000002582. 10.1227/neu.0000000000002582 PMC12245298 37409817

[61] Chesnut RM. Pediatric Severe Traumatic Brain Injury in Latin America - A Randomized Trial Comparing Two Management Protocols [Internet]. clinicaltrials.gov; 2025 June. Report No.: NCT05566431. https://clinicaltrials.gov/study/NCT05566431. Accessed 9 May 2026.

