# Supplementary material for: Impacts of intracranial pressure monitoring on mortality and length of stay in severe traumatic brain injury: a systematic review and meta-analysis
Source: Eur J Trauma Emerg Surg. 2026 Aug 12;52(1):249. doi: 10.1007/s00068-026-03302-5 (PMC13468859; doi:10.1007/s00068-026-03302-5)
Supplement: Supplementary file 1 [file 68_2026_3302_MOESM1_ESM.pdf]

Supplementary Digital Content 2. Search strategy

SDC 2a. MEDLINE/PubMed search strategy

Database search strategy: MEDLINE/PubMed
#1. ((brain[ti] OR intracranial[ti] OR head[ti] OR craniocerebral[ti]) AND (trauma*[ti] OR injury[ti] OR injuries[ti])) OR "TBI"[ti] OR "TBI-induced"[ti] OR "traumatic encephalopathy"[ti]<br><br>#2. ((brain[tiab] OR intracranial[tiab] OR head[tiab] OR craniocerebral[tiab]) AND (trauma*[tiab] OR injury[tiab] OR injuries[tiab])) OR "TBI"[tiab] OR "TBI-induced"[tiab] OR "traumatic encephalopathy"[tiab]<br><br>#3. #2 NOT #1<br><br>#4. "brain injuries, traumatic"[mh] OR "brain hemorrhage, traumatic"[mh] OR "brain contusion"[mh]<br><br>#5. pressure[ti] OR "ICP"[ti] OR "intracranial hypertension"[ti]<br><br>#6. (("ICP"[ti] OR pressure[ti] OR intraparenchymal[ti] OR "cerebrospinal fluid"[ti] OR "CSF"[ti] OR "External Ventricular Drain"[ti] OR "EVD"[ti]) AND (monitor*[ti] OR measur*[ti])) OR "Subarachnoid Bolt"[ti] OR "Epidural bolt"[ti] OR sensor[ti] OR "Wireless telemetering"[ti] OR "ventriculostomy EDMS"[ti] OR ((fiberoptic[ti] OR "fibre optic"[ti]) AND (catheter[ti] OR monitor[ti]))<br><br>#7. (("ICP"[tiab] OR pressure[tiab] OR intraparenchymal[tiab] OR "cerebrospinal fluid"[tiab] OR "CSF"[tiab] OR "External Ventricular Drain"[tiab] OR "EVD"[tiab]) AND (monitor*[tiab] OR measur*[tiab])) OR "Subarachnoid Bolt"[tiab] OR "Epidural bolt"[tiab] OR sensor[tiab] OR "Wireless telemetering"[tiab] OR "ventriculostomy EDMS"[tiab] OR ((fiberoptic[tiab] OR "fibre optic"[tiab]) AND (catheter[tiab] OR monitor[tiab]))<br><br>#8. #7 NOT #6<br><br>#9. #1 AND #6<br><br>#10. #3 AND #8 AND #5<br><br>#11. #2 AND #4 AND #5<br><br>#12. #9 OR #10 OR #11<br><br>#13. outcome*[tiab] OR mortalit*[tiab] OR morbidit*[tiab] OR "length of stay"[tiab] OR "LOS"[tiab] OR "days to discharge"[tiab]<br><br>#14. dogs[ti] OR dog[ti] OR animal*[ti] OR rat[ti] OR rats[ti] OR mouse[ti] OR mice[ti] OR cat[ti] OR cats[ti] OR feline[ti] OR porcine[ti] OR bovine[ti] OR cow[ti] OR primate*[ti] OR rabbit[ti] OR rabbits[ti] OR invitro[ti] OR "in vitro"[ti] OR monkey[ti] OR monkeys[ti] OR equine[ti] OR horse[ti] OR horses[ti] OR frog[ti] OR frogs[ti] OR "in-vitro"[ti] OR cadaver*[ti] OR adolescent[ti] OR teenager[ti] OR baby[ti] OR babies[ti] OR neonate*[ti] OR infant*[ti] OR child*[ti] OR toddler*[ti] OR "aged"[mh] OR old*[ti] OR elder*[ti] OR geriatric*[ti] OR "aged, 80 and over"[mh] OR "frail elderly"[mh] OR ((death[ti] OR morta*[ti]) AND ("ED"[ti] OR "emergency department"[ti] OR "accident and emergency"[ti] OR "A&E"[ti]))<br><br>#15. #12 AND #13 NOT #14

Field definitions: All fields [ALL], Title [TI], Title and Abstract [TIAB], MeSH Term [MH], MeSH Term Unexploded [Mesh:noexp]

SDC 2b. Embase search strategy

Database search strategy: Embase
#1. (((brain OR intracranial OR head OR craniocerebral) NEAR/3 (trauma* OR injury OR injuries)):ti) OR tbi:ti OR 'tbi induced':ti OR 'traumatic encephalopathy':ti<br><br>#2. ((brain OR intracranial OR head OR craniocerebral) NEAR/3 (trauma* OR injury OR injuries)):ti,ab OR tbi:ti,ab OR 'tbi induced':ti,ab OR 'traumatic encephalopathy':ti,ab<br><br>#3. #2 NOT #1<br><br>#4. 'traumatic brain injury'/exp OR 'brain contusion'/exp<br><br>#5. pressure:ti OR ICP:ti OR 'intracranial hypertension':ti<br><br>#6. ((ICP OR pressure OR intraparenchymal OR 'cerebrospinal fluid' OR CSF OR 'External Ventricular Drain' OR EVD) NEAR/3 (monitor* OR measur*)):ti OR 'Subarachnoid Bolt':ti OR 'Epidural bolt':ti OR sensor:ti OR 'Wireless telemetering':ti OR 'ventriculostomy EDMS':ti OR ((fiberoptic OR 'fibre optic') NEAR/3 (catheter OR monitor)):ti<br><br>#7. (('ICP':ti,ab OR pressure:ti,ab OR intraparenchymal:ti,ab OR 'cerebrospinal fluid':ti,ab OR 'CSF':ti,ab OR 'External Ventricular Drain':ti,ab OR 'EVD':ti,ab) AND (monitor*:ti,ab OR measur*:ti,ab)) OR 'Subarachnoid Bolt':ti,ab OR 'Epidural bolt':ti,ab OR sensor:ti,ab OR 'Wireless telemetering':ti,ab OR 'ventriculostomy EDMS':ti,ab OR ((fiberoptic:ti,ab OR 'fibre optic':ti,ab) AND (catheter:ti,ab OR monitor:ti,ab))<br><br>#8. #7 NOT #6<br><br>#9. #1 AND #6<br><br>#10. #3 AND #8 AND #5<br><br>#11. #2 AND #4 AND #5<br><br>#12. #9 OR #10 OR #11<br><br>#13. outcome*:ti,ab OR mortalit*:ti,ab OR morbidit*:ti,ab OR 'length of stay':ti,ab OR LOS:ti,ab OR 'days to discharge':ti,ab<br><br>#14. dogs:ti OR dog:ti OR animal*:ti OR rat:ti OR rats:ti OR mouse:ti OR mice:ti OR cat:ti OR cats:ti OR feline:ti OR porcine:ti OR bovine:ti OR cow:ti OR primate*:ti OR rabbit:ti OR rabbits:ti OR invitro:ti OR 'in vitro':ti OR monkey:ti OR monkeys:ti OR equine:ti OR horse:ti OR horses:ti OR frog:ti OR frogs:ti OR 'in-vitro':ti OR cadaver*:ti OR adolescent:ti OR teenager:ti OR baby:ti OR babies:ti OR neonate*:ti OR infant*:ti OR child*:ti OR toddler*:ti OR aged/exp OR old*:ti OR elder*:ti OR geriatric*:ti OR 'aged, 80 and over'/exp OR 'frail elderly'/exp OR ((death:ti OR morta*:ti) AND (ED:ti OR 'emergency department':ti OR 'accident and emergency':ti OR 'A&E':ti))<br><br>#15. #12 AND #13 NOT #14

Field definitions: Article title :ti, abstract :ab, index term /de, drug trade name :tn, exploded index term /exp

SDC 2c. Cochrane Central Register of Controlled Trials search strategy

Database search strategy: Cochrane Central Register of Controlled Trials
#1. ((brain:ti OR intracranial:ti OR head:ti OR craniocerebral:ti) AND (trauma*:ti OR injury:ti OR injuries:ti)) OR TBI:ti OR TBI-induced:ti OR "traumatic encephalopathy":ti<br><br>#2. ((brain:ti,ab OR intracranial:ti,ab OR head:ti,ab OR craniocerebral:ti,ab) AND (trauma*:ti,ab OR injury:ti,ab OR injuries:ti,ab)) OR TBI:ti,ab OR TBI-induced:ti,ab OR "traumatic encephalopathy":ti,ab<br><br>#3. #2 NOT #1<br><br>#4. [mh "brain injuries, traumatic"] OR [mh "brain hemorrhage, traumatic"] OR [mh "brain contusion"]<br><br>#5. pressure:ti OR ICP:ti OR "intracranial hypertension":ti<br><br>#6. ((ICP:ti OR pressure:ti OR intraparenchymal:ti OR "cerebrospinal fluid":ti OR CSF:ti OR "External Ventricular Drain":ti OR EVD:ti) AND (monitor*:ti OR measur*:ti)) OR "Subarachnoid Bolt":ti OR "Epidural bolt":ti OR sensor:ti OR "Wireless telemetering":ti OR "ventriculostomy EDMS":ti OR ((fiberoptic:ti OR "fibre optic":ti) AND (catheter:ti OR monitor:ti))<br><br>#7. ((ICP:ti,ab OR pressure:ti,ab OR intraparenchymal:ti,ab OR "cerebrospinal fluid":ti,ab OR CSF:ti,ab OR "External Ventricular Drain":ti,ab OR EVD:ti,ab) AND (monitor*:ti,ab OR measur*:ti,ab)) OR "Subarachnoid Bolt":ti,ab OR "Epidural bolt":ti,ab OR sensor:ti,ab OR "Wireless telemetering":ti,ab OR "ventriculostomy EDMS":ti,ab OR ((fiberoptic:ti,ab OR "fibre optic":ti,ab) AND (catheter:ti,ab OR monitor:ti,ab))<br><br>#8. #7 NOT #6<br><br>#9. #1 AND #6<br><br>#10. #3 AND #8 AND #5<br><br>#11. #2 AND #4 AND #5<br><br>#12. #9 OR #10 OR #11<br><br>#13. outcome*:ti,ab OR mortalit*:ti,ab OR morbidit*:ti,ab OR "length of stay":ti,ab OR LOS:ti,ab OR "days to discharge":ti,ab<br><br>#14. dogs:ti OR dog:ti OR animal*:ti OR rat:ti OR rats:ti OR mouse:ti OR mice:ti OR cat:ti OR cats:ti OR feline:ti OR porcine:ti OR bovine:ti OR cow:ti OR primate*:ti OR rabbit:ti OR rabbits:ti OR invitro:ti OR "in vitro":ti OR monkey:ti OR monkeys:ti OR equine:ti OR horse:ti OR horses:ti OR frog:ti OR frogs:ti OR "in-vitro":ti OR cadaver*:ti OR adolescent:ti OR teenager:ti OR baby:ti OR babies:ti OR neonate*:ti OR infant*:ti OR child*:ti OR toddler*:ti OR [mh aged] OR old*:ti OR elder*:ti OR geriatric*:ti OR [mh "aged, 80 and over"] OR [mh "frail elderly"] OR ((death:ti OR morta*:ti) AND (ED:ti OR "emergency department":ti OR "accident and emergency":ti OR "A&E":ti))<br><br>#15. #12 AND #13 NOT #14

SDC 2d. Integrated discovery-layer search strategy

Database search strategy for institutional integrated search interface / EBSCO EDS discovery layer additional databases.
S1 (((TI brain) OR (TI intracranial) OR (TI head) OR (TI craniocerebral)) AND ((TI trauma*) OR (TI injury) OR (TI injuries))) OR (TI TBI) OR (TI TBI-induced) OR (TI "traumatic encephalopathy")<br><br>S2 (((TI brain OR AB brain) OR (TI intracranial OR AB intracranial) OR (TI head OR AB head) OR (TI craniocerebral OR AB craniocerebral)) AND ((TI trauma* OR AB trauma*) OR (TI injury OR AB injury) OR (TI injuries OR AB injuries))) OR (TI TBI OR AB TBI) OR (TI TBI-induced OR AB TBI-induced) OR (TI "traumatic encephalopathy" OR AB "traumatic encephalopathy")<br><br>S3 S2 NOT S1<br><br>S4 (SU "brain injuries, traumatic") OR (SU "brain hemorrhage, traumatic") OR (SU "brain contusion")<br><br>S5 (TI pressure) OR (TI ICP) OR (TI "intracranial hypertension")<br><br>S6 (((TI ICP) OR (TI pressure) OR (TI intraparenchymal) OR (TI "cerebrospinal fluid") OR (TI CSF) OR (TI "External Ventricular Drain") OR (TI EVD)) AND ((TI monitor*) OR (TI measur*))) OR (TI "Subarachnoid Bolt") OR (TI "Epidural bolt") OR (TI sensor) OR (TI "Wireless telemetering") OR (TI "ventriculostomy EDMS") OR (((TI fiberoptic) OR (TI "fibre optic")) AND ((TI catheter) OR (TI monitor)))<br><br>S7 (((TI ICP OR AB ICP) OR (TI pressure OR AB pressure) OR (TI intraparenchymal OR AB intraparenchymal) OR (TI "cerebrospinal fluid" OR AB "cerebrospinal fluid") OR (TI CSF OR AB CSF) OR (TI "External Ventricular Drain" OR AB "External Ventricular Drain") OR (TI EVD OR AB EVD)) AND ((TI monitor* OR AB monitor*) OR (TI measur* OR AB measur*))) OR (TI "Subarachnoid Bolt" OR AB "Subarachnoid Bolt") OR (TI "Epidural bolt" OR AB "Epidural bolt") OR (TI sensor OR AB sensor) OR (TI "Wireless telemetering" OR AB "Wireless telemetering") OR (TI "ventriculostomy EDMS" OR AB "ventriculostomy EDMS") OR (((TI fiberoptic OR AB fiberoptic) OR (TI "fibre optic" OR AB "fibre optic")) AND ((TI catheter OR AB catheter) OR (TI monitor OR AB monitor)))<br><br>S8 S7 NOT S6<br><br>S9 S1 AND S6<br><br>S10 S3 AND S8 AND S5<br><br>S11 S2 AND S4 AND S5<br><br>S12 S9 OR S10 OR S11<br><br>S13 (TI outcome* OR AB outcome*) OR (TI mortalit* OR AB mortalit*) OR (TI morbidit* OR AB morbidit*) OR (TI "length of stay" OR AB "length of stay") OR (TI LOS OR AB LOS) OR (TI "days to discharge" OR AB "days to discharge")<br><br>S14 (TI dogs) OR (TI dog) OR (TI animal*) OR (TI rat) OR (TI rats) OR (TI mouse) OR (TI mice) OR (TI cat) OR (TI cats) OR (TI feline) OR (TI porcine) OR (TI bovine) OR (TI cow) OR (TI primate*) OR (TI rabbit) OR (TI rabbits) OR (TI invitro) OR (TI "in vitro") OR (TI monkey) OR (TI monkeys) OR (TI equine) OR (TI horse) OR (TI horses) OR (TI frog) OR (TI frogs) OR (TI "in-vitro") OR (TI cadaver*) OR (TI adolescent) OR (TI teenager) OR (TI baby) OR (TI babies) OR (TI neonate*) OR (TI infant*) OR (TI child*) OR (TI toddler*) OR (MH aged+) OR (TI old*) OR (TI elder*) OR (TI geriatric*) OR (MH "aged, 80 and over+") OR (MH "frail elderly+") OR (((TI death) OR (TI morta*)) AND ((TI ED) OR (TI "emergency department") OR (TI "accident and emergency") OR (TI "A&E")))<br><br>S15 S12 AND S13 NOT S14

Limit: Peer reviewed journals

Databases searched:

Academic Search Index

AccessScience

BioOne Complete

BMJ Best Practice

BMJ Clinical Evidence

CINAHL Complete

ClinicalTrials.gov

Cochrane Database of Systematic Reviews

CogPrints

Complementary Index

Directory of Open Access Journals

Emerald Insight

ERIC

Harvard Library Bibliographic Database

Informit Health Collection

Informit Indigenous Collection

McGraw-Hill Medical

MEDLINE Complete

Minority Health Archive

Oxford Medicine Online

Oxford Reference

R2 Digital Library

Rural and Remote Health Database

ScienceDirect
