# Supplementary material for: Impacts of intracranial pressure monitoring on mortality and length of stay in severe traumatic brain injury: a systematic review and meta-analysis
Source: Eur J Trauma Emerg Surg. 2026 Aug 12;52(1):249. doi: 10.1007/s00068-026-03302-5 (PMC13468859; doi:10.1007/s00068-026-03302-5)
Supplement: Supplementary file 2 [file 68_2026_3302_MOESM2_ESM.pdf]

Supplementary Digital Content 3. Confounding control in observational studies

Study & Year	Age	GCS	Pupils	Injury severity	Comorbidity / physiology	Radiological severity	Additional adjusted factors	Main confounding limitation
Agrawal et al. 2015	●	●	–	●	●	●	Posturing, hypotension, CT abnormality	Reasonable adjustment, but observational design and incomplete long-term follow-up remain limitations
Aiolfi et al. 2017	●	●	–	●	●	–	Need for craniectomy	Registry-based adjustment without full neuroseverity granularity
Alali et al. 2013	●	●	●	●	●	●	Mechanism, insurance, teaching status, bed number	Administrative dataset with limited physiological granularity
Alali et al. 2015	●	●	–	●	●	–	–	Administrative paediatric dataset with limited imaging and physiological granularity
Al-Anazi et al. 2004	–	–	–	–	–	–	–	No meaningful confounding control
Alkhoury et al. 2014	●	●	–	●	–	●	–	Registry-based paediatric analysis with limited physiological detail
Al Saiegh et al. 2020	●	●	–	●	–	–	Craniotomy	Discordant models and likely residual confounding
Biersteker et al. 2012	●	●	●	●	●	●	Hypotension, hypoxia, extracranial injury	Residual confounding by indication remains possible despite adjustment
Dawes et al. 2015	●	●	–	●	●	●	Subdural haematoma, intraparenchymal contusion, mass effect, INR	Residual confounding remains possible despite propensity matching
Farahvar et al. 2012	●	●	●	–	●	●	Hypotension	Restricted treated cohort; not representative of all severe TBI
Foote et al. 2022	–	●	–	–	–	●	SAH, SDH	No adjusted analysis; strong confounding by indication likely
Fu et al. 2020	●	●	–	–	–	●	Contusion volume, neurological deterioration	Matching restricted to contusion-predominant TBI; residual confounding possible
Gao et al. 2013	–	–	–	–	–	–	–	No formal adjusted analysis; highly selected operative subgroup
Griesdale et al. 2010	●	●	–	●	●	●	Craniotomy, SAH, DAI, SDH, mannitol, jugular bulb catheter, hypothermia	Residual confounding by treatment intensity and indication likely
Haddad et al. 2011	●	●	–	–	●	–	APACHE II, postoperative status	Clinician-directed monitor placement likely introduced confounding by indication
Jitchanvichai et al. 2024	●	●	–	–	●	●	Blood pressure, basal cistern obliteration, midline shift	Low ICP utilisation and likely strong treatment selection
Kostić et al. 2011	–	–	–	–	–	–	–	No meaningful multivariable confounding control; very small quasi-randomised study
Lane et al. 2000	–	–	–	●	–	–	MAIS head, injury type (blunt vs penetrating)	GCS excluded from modelling; substantial residual confounding likely
Lele et al. 2019	●	●	–	●	●	●	Polytrauma, transfers, mechanical ventilation duration	Residual confounding and survivor-selection bias possible
Mauritz et al. 2008	●	●	–	●	●	–	SAPS II, admission type, vasopressor use, hospital size	No pupil or CT severity adjustment; residual confounding remains

**Nattino et al. 2023**	●	●	–	●	●	●	Sex	Stronger confounding control than most studies, but residual confounding remains possible
**Peled et al. 2025**	●	●	–	●	–	–	AIS severity strata, age strata	Registry-based analysis with heterogeneous age groups and likely residual confounding
**Robba et al. 2021**	●	●	●	●	●	–	Centre effects, mixed acute brain injury cohort	Mixed pathology cohort limits exchangeability for TBI-specific inference
**Schupper et al. 2019**	●	●	–	●	–	●	Insurance, number of neurosurgeons at centre	Study focus was predictor modelling for monitor use rather than robust causal adjustment
**Shafi et al. 2008**	●	●	–	●	●	●	Craniotomy, blood pressure on arrival, mechanism, associated injuries	Registry-based analysis with strong confounding by indication
**Suehiro et al. 2017**	●	●	–	●	–	●	Treatment intensity differences across centres	Marked treatment imbalance and no strict cross-centre ICP indication
**Talving et al. 2013**	●	●	●	●	●	●	Coagulation profile, nutrition, craniotomy	Single-centre observational design; residual confounding likely
**Yang et al. 2022**	●	●	–	●	–	–	–	Residual confounding remains despite matching; no long-term functional adjustment
**You et al. 2016**	●	–	–	●	●	–	–	No formal adjusted comparative model; strong selection bias risk
**Zeng et al. 2013**	●	●	–	●	●	●	Mannitol exposure	Non-randomised design; treatment variables may also act as post-exposure mediators

NB: ● indicates that the variable was explicitly included in multivariable adjustment, propensity score modelling, matching, or weighting, as reported by the original study. “Injury severity” includes measures such as AIS, ISS, extracranial trauma burden, polytrauma, or related injury-severity covariates. “Comorbidity / physiology” includes variables such as APACHE/SAPS scores, hypotension, hypoxia, vasopressor use, coagulation profile, baseline medical status, mechanical ventilation, or other physiological variables. “Radiological severity” includes abnormal CT classification, mass effect, midline shift, basal cistern status, contusion burden, traumatic SAH/SDH, DAI, or other intracranial imaging features. This table summarizes confounding control in observational studies only; the included randomized controlled trial was assessed separately using RoB 2.0 in Supplementary Digital Content 4.

Abbreviations: AIS, Abbreviated Injury Scale; APACHE, Acute Physiology and Chronic Health Evaluation; CT, computed tomography; DAI, diffuse axonal injury; GCS, Glasgow Coma Scale; ICP, intracranial pressure; INR, international normalized ratio; ISS, Injury Severity Score; MAIS, Maximum Abbreviated Injury Scale; PS, propensity score; PSM, propensity score matching; SAH, subarachnoid hemorrhage; SAPS, Simplified Acute Physiology Score; SDH, subdural hematoma; TBI, traumatic brain injury.
