# Supplementary material for: Impacts of intracranial pressure monitoring on mortality and length of stay in severe traumatic brain injury: a systematic review and meta-analysis
Source: Eur J Trauma Emerg Surg. 2026 Aug 12;52(1):249. doi: 10.1007/s00068-026-03302-5 (PMC13468859; doi:10.1007/s00068-026-03302-5)
Supplement: Supplementary file 3 [file 68_2026_3302_MOESM3_ESM.pdf]

Supplementary Digital Content 4. Quality assessment

Supplementary Digital Content 4a. CEBM Level of Evidence assessment

Study & Year	Country	Study Design	Level of Evidence (CEBM)
**Agrawal et al. 2015**	India	Prospective registry with retrospective adjusted cohort analysis	2
**Aiolfi et al. 2017**	USA	Retrospective registry cohort	3
**Alali et al. 2013**	USA / Canada	Retrospective multicentre observational cohort	3
**Alali et al. 2015**	USA	Retrospective multicentre observational cohort	3
**Al-Anazi et al. 2004**	Saudi Arabia	Single-centre retrospective cohort	3
**Alkhoury et al. 2014**	USA	Retrospective multicentre registry cohort	3
**Al Saiegh et al. 2020**	USA	Retrospective statewide registry cohort	3
**Biersteker et al. 2012**	Netherlands	Prospective multicentre observational cohort	2
**Chesnut et al. 2012**	Bolivia & Ecuador	Multicentre randomised controlled trial	1
**Dawes et al. 2015**	USA	Prospective multicentre observational cohort with propensity score matching	2
**Farahvar et al. 2012**	USA	Prospective multicentre observational cohort	2
**Foote et al. 2022**	USA	Single-centre retrospective observational cohort	3
**Fu et al. 2020**	China	Retrospective multicentre cohort with propensity score matching	3
**Gao et al. 2013**	China	Retrospective cohort (operative subgroup)	3
**Griesdale et al. 2010**	Canada	Retrospective single-centre cohort with propensity-adjusted multivariable regression	3
**Haddad et al. 2011**	Saudi Arabia	Single-centre retrospective cohort	3
**Jitchanvichai et al. 2024**	Thailand	Single-centre retrospective cohort with propensity score matching	3
**Kostić et al. 2011**	Serbia	Prospective quasi-randomised comparative study	2
**Lane et al. 2000**	Canada	Retrospective observational cohort / trauma registry study	3
**Lele et al. 2019**	India	Prospective observational cohort with IPW / multivariable regression	2

**Mauritz et al. 2008**	Austria	Prospective multicentre cohort	2
**Nattino et al. 2023**	Italy & Hungary	Prospective multicentre observational cohort with full matching propensity analysis	2
**Peled et al. 2025**	Israel	Retrospective registry cohort	3
**Robba et al. 2021**	International	Prospective multicentre observational cohort with IPTW	2
**Schupper et al. 2019**	USA	Retrospective registry cohort with propensity methods	3
**Shafi et al. 2008**	USA	Retrospective multicentre registry cohort	3
**Suehiro et al. 2017**	Japan	Retrospective multicentre registry cohort	3
**Talving et al. 2013**	USA	Prospective observational cohort	2
**Yang et al. 2022**	China	Prospective multicentre observational cohort	2
**You et al. 2016**	China	Prospective single-centre observational cohort	2
**Zeng et al. 2013**	China	Prospective controlled observational study	2

NB: Levels of evidence were assigned using the Oxford Centre for Evidence-Based Medicine framework, with randomised controlled trials classified as Level 1, prospective comparative observational studies as Level 2, and retrospective comparative cohort or registry studies as Level 3.
Abbreviations: CEBM, Centre for Evidence-Based Medicine; IPW, inverse probability weighting; IPTW, inverse probability of treatment weighting.

Supplementary Digital Content 4b. STROBE reporting assessment of observational studies

Study & Year	Study Design	STROBE items absent (n/22)	STROBE items absent (%)	STROBE reporting quality	Key reporting limitations
**Agrawal et al. 2015**	Prospective registry with retrospective adjusted cohort analysis	2	9.1%	Good	Generalisability not clearly addressed; funding information not clearly reported
**Aiolfi et al. 2017**	Retrospective registry cohort	1	4.5%	Good	Funding information absent
**Alali et al. 2013**	Retrospective multicentre observational cohort	1	4.5%	Good	Funding information absent
**Alali et al. 2015**	Retrospective multicentre observational cohort with multilevel modelling + propensity-matched sensitivity analysis	2	9.1%	Good	Funding information not clearly reported; limited reporting of longer-term outcome availability
**Al-Anazi et al. 2004**	Single-centre retrospective cohort	4	18.2%	Limited	Data sources / measurement absent; bias handling absent; generalisability absent; funding information absent
**Alkhoury et al. 2014**	Retrospective multicentre registry cohort	0	0%	Good	Additional adjusted analyses limited
**Al Saiegh et al. 2020**	Retrospective statewide registry cohort	0	0%	Good	No major reporting deficiencies
**Biersteker et al. 2012**	Prospective multicentre observational cohort	0	0%	Good	No major reporting deficiencies
**Dawes et al. 2015**	Retrospective cohort with propensity score matching	0	0%	Good	No major reporting deficiencies
**Farahvar et al. 2012**	Prospective multicentre observational cohort	1	4.5%	Good	Bias domain not clearly addressed in detail
**Foote et al. 2022**	Single-centre retrospective observational cohort	1	4.5%	Fair	Other analyses incomplete; generalisability present but did not meet criteria
**Fu et al. 2020**	Retrospective multicentre cohort with propensity score matching	1	4.5%	Good	Funding information absent

**Gao et al. 2013**	Retrospective cohort (operative subgroup)	2	9.1%	Fair	Limited reporting of bias and generalisability; highly selected operative subgroup
**Griesdale et al. 2010**	Retrospective single-centre cohort with propensity-adjusted multivariable regression	1	4.5%	Good	Funding information absent
**Haddad et al. 2011**	Single-centre retrospective cohort	1	4.5%	Good	Funding information absent
**Jitchanvichai et al. 2024**	Single-centre retrospective cohort with propensity score matching	1	4.5%	Good	Data sources / measurement not fully reported
**Kostić et al. 2011**	Prospective quasi-randomised comparative study	2	9.1%	Fair	Bias reporting absent; quantitative variables reporting incomplete; generalisability present but did not meet criteria
**Lane et al. 2000**	Retrospective observational cohort / trauma registry study	1	4.5%	Good	Funding information absent
**Lele et al. 2019**	Prospective observational cohort with IPW / multivariable regression	2	9.1%	Good	Funding information not clearly reported; some data-source / measurement detail limited
**Mauritz et al. 2008**	Prospective multicentre cohort	2	9.1%	Fair	Data sources / measurement and bias handling incompletely reported
**Nattino et al. 2023**	Prospective multicentre observational cohort with full matching propensity analysis	0	0%	Good	No major reporting deficiencies
**Robba et al. 2021**	Prospective multicentre observational cohort with IPTW	1	4.5%	Good	Generalisability not clearly addressed
**Schupper et al. 2019**	Retrospective registry cohort with propensity methods	0	0%	Fair	Data sources / measurement present but did not meet criteria
**Shafi et al. 2008**	Retrospective multicentre registry cohort	1	4.5%	Good	Funding information absent
**Talving et al. 2013**	Prospective observational cohort	1	4.5%	Good	Funding information absent
**You et al. 2016**	Prospective single-centre observational cohort	1	4.5%	Good	Generalisability not clearly addressed
**Zeng et al. 2013**	Prospective controlled observational study	1	4.5%	Good	Generalisability not clearly addressed

Key: Good: 0–2 absent items, with no major reporting domain failure; Fair: 0–2 absent items with at least one important item present but not meeting criteria, or 3–5 absent items; Limited: major reporting deficiencies across key methodological or discussion domains

NB: This summary was derived from the completed item-by-item STROBE matrix and extended to the remaining included observational studies using the same decision logic. "STROBE items absent" refers to checklist items that were absent / not assessable. Good = most STROBE items present and meeting criteria, with only minor omissions; Fair = several omissions and/or at least one key item present but not meeting criteria; Limited = major reporting deficiencies across important methodological or discussion domains.

Abbreviations: IPW, inverse probability weighting; IPTW, inverse probability of treatment weighting; PSM, propensity score matching; STROBE, Strengthening the Reporting of Observational Studies in Epidemiology.

Supplementary Digital Content 4c. Risk of Bias 2.0 assessment of the included randomized controlled trial

**Study & Year**	**Outcome**	*D1. Randomisation process*	*D2. Deviations from intended interventions*	*D3. Missing outcome data*	*D4. Measurement of the outcome*	*D5. Selection of the reported result*	**Overall RoB 2.0 judgement**
**Chesnut et al. 2012**	Mortality	Low risk 🟢	Low risk 🟢	Low risk 🟢	Low risk 🟢	Low risk 🟢	**Low risk** 🟢
	GOS-E	Low risk 🟢	Low risk 🟢	Low risk 🟢	Low risk 🟢	Low risk 🟢	**Low risk** 🟢
	ICU Length of stay	Low risk 🟢	Low risk 🟢	Low risk 🟢	Low risk 🟢	Low risk 🟢	**Low risk** 🟢

Legend: 🟢 Low risk; 🟡 Some concerns; 🔴 High risk

NB: Risk of bias was assessed using the Cochrane Risk of Bias 2.0 tool for randomized trials. The included randomized controlled trial was assessed across the relevant outcome domains extracted in this review, including mortality, GOS-E, and ICU length of stay. Overall risk of bias was judged according to the RoB 2.0 domain-based algorithm.

Abbreviations: D1, bias arising from the randomization process; D2, bias due to deviations from intended interventions; D3, bias due to missing outcome data; D4, bias in measurement of the outcome; D5, bias in selection of the reported result; GOS-E, Glasgow Outcome Scale–Extended; ICU, intensive care unit; RoB, risk of bias.
