# Supplementary material for: Impacts of intracranial pressure monitoring on mortality and length of stay in severe traumatic brain injury: a systematic review and meta-analysis
Source: Eur J Trauma Emerg Surg. 2026 Aug 12;52(1):249. doi: 10.1007/s00068-026-03302-5 (PMC13468859; doi:10.1007/s00068-026-03302-5)
Supplement: Supplementary file 4 [file 68_2026_3302_MOESM4_ESM.pdf]

Supplementary Digital Content 5. Excluded studies of interest

Authors & Year	Title	Primary reason for exclusion
**Aiolfi et al. 2018**	Intracranial Pressure Monitoring in Severe Blunt Head Trauma: Does the Type of Monitoring Device Matter?	Comparator was monitor device type among monitored patients; overlapping TQIP device-subset analysis rather than ICP-monitored versus unmonitored comparison.
**Banik et al. 2019**	Intracranial Pressure Monitoring in Children with Severe Traumatic Brain Injury	Paediatric cohort only.
**Beach et al. 2025**	The Impact of a Protocolization Approach to Increase the Use of and Timeliness to Intracranial Pressure Monitoring in Patients With Severe Traumatic Brain Injury at a Level 1 Trauma Center	Assessed protocolisation and timeliness of ICP monitor placement rather than outcomes in ICP-monitored versus unmonitored patients.
**Dolmans et al. 2023**	External Ventricular Drains versus Intraparenchymal Pressure Monitors in Moderate to Severe Traumatic Brain Injury	Comparator was monitor device type (EVD vs IPM), not ICP-monitored versus unmonitored patients.
**Greil et al. 2025**	External Ventricular Drain Versus Intraparenchymal Pressure Monitor in Severe Traumatic Brain Injury: A TRACK-TBI Study	Comparator was monitor device type (EVD vs IPM), not ICP-monitored versus unmonitored patients.
**Kim et al. 2014**	Significance of Intracranial Pressure Monitoring after Early Decompressive Craniectomy in Patients with Severe Traumatic Brain Injury	Restricted to patients undergoing early decompressive craniectomy; evaluated postoperative ICP monitoring within a surgical subset rather than an unselected severe TBI cohort.
**Li et al. 2025**	Impact of Intracranial Pressure Monitoring on Six-month Neurologic Outcomes in Severe Traumatic Brain Injury: A Retrospective Cohort Study	Restricted to adults with severe TBI undergoing primary decompressive craniectomy; evaluated postoperative ICP monitoring within a surgical subset rather than an unselected severe TBI cohort.
**Mauritz et al. 2007**	Severe Traumatic Brain Injury in Austria IV: Intensive Care Management	Did not provide the monitored versus unmonitored comparative outcome structure required for the review's primary analyses.
**Moyer et al. 2023**	Impact of early external ventricular drainage on functional outcome after traumatic brain injury: a bicentric retrospective cohort analysis	Comparator was timing of EVD insertion among patients who all received EVD, rather than ICP-monitored versus unmonitored patients.
**Piccinini et al. 2017**	Intracranial pressure monitoring after primary decompressive craniectomy in traumatic brain injury: a clinical study	Restricted to patients undergoing primary decompressive craniectomy; evaluated postoperative ICP monitoring within a surgical subset rather than an unselected severe TBI cohort.
**Shibahashi et al. 2024**	Intracranial Pressure Monitoring in Children With Severe Traumatic Brain Injury	Paediatric cohort only.
**Thompson et al. 2008**	Evaluation of the Effect of Intensity of Care on Mortality After Traumatic Brain Injury	Wrong primary question; evaluated intensity of care and likelihood of receiving ICP monitoring rather than comparative outcomes for ICP-monitored versus unmonitored patients.

NB: This table includes full-text articles considered potentially relevant during screening but excluded from the final review. Only studies not included in Table 1 are listed. Where multiple exclusion reasons applied, the primary reason for exclusion is shown.
Abbreviations: EVD, external ventricular drain; ICP, intracranial pressure; ICPm, intracranial pressure monitoring; IPM, intraparenchymal pressure monitor; TBI, traumatic brain injury; TQIP, Trauma Quality Improvement Program.
