# Supplementary material for: Impacts of intracranial pressure monitoring on mortality and length of stay in severe traumatic brain injury: a systematic review and meta-analysis
Source: Eur J Trauma Emerg Surg. 2026 Aug 12;52(1):249. doi: 10.1007/s00068-026-03302-5 (PMC13468859; doi:10.1007/s00068-026-03302-5)
Supplement: Supplementary file 5 [file 68_2026_3302_MOESM5_ESM.pdf]

SDC 6 – Descriptive study tables

SDC 6a. Population characteristics and demographic details of included studies

SDC 6b. GOS and GOS-E/GOSE outcomes

SDC 6c. Functional outcomes in ICP-monitored versus non-ICP-monitored groups: descriptive per-study summary

SDC 6d. Functional outcomes in ICP-monitored versus non-ICP-monitored groups: summary by outcome measure

SDC 6e. FIM outcomes

SDC 6f. Favorable versus unfavorable functional outcomes

SDC 6g. Complications and treatment burden in ICP-monitored versus non-ICP-monitored groups

SDC 6a. Population characteristics and demographic details of included studies

Study & Year (Study period)	Country	Study Design	n	Population (Age (y), % Male)		Inclusion Criteria	Type of monitoring	Summary of key findings
				ICPm	No ICPm			
**Agrawal et al. 2015 (Jun 2010–Jul 2012)**	India	Prospective registry with retrospective propensity score-adjusted cohort analysis	1,345	32 (25–45); 89% male	32 (25–45); 89% male	Severe TBI: GCS ≤8, age >12 y, meeting BTF ICPm criteria; abnormal CT or normal CT with ≥2 of age >40 y, SBP <90 mmHg, or posturing; excluded death <24 h	Intraparenchymal monitor; EVD reserved for hydrocephalus	ICPm was associated with lower in-hospital mortality, but 6-month functional outcome was not significantly improved; major caveat: observational design with incomplete longer-term follow-up.
**Aiolfi et al. 2017 (2013–2014)**	USA	TQIP registry; retrospective cohort	13,188	41 (25–56); 76.7% male	54 (33–72); 70.4% male	Isolated severe blunt TBI meeting BTF criteria: GCS <9 with abnormal CT, or normal CT with ≥2 of age >40 y, motor posturing, or SBP <90 mmHg; age ≥16 y; excluded extracranial AIS ≥3, interhospital transfers, and dead on arrival	Mixed / not specified	No mortality benefit was shown and functional independence was worse with ICPm; major caveat: strong confounding by indication in registry data.
**Alali et al. 2013 (2009–2011)**	USA / Canada	ACS TQIP registry; retrospective multicentre observational cohort with multilevel modelling	10,628	43 (26–56); approximately 70–78% male	53 (31–72); approximately 70–78% male	Adults ≥16 y with severe TBI, GCS ≤8, and acute intracranial lesion; excluded penetrating injury, polytrauma, AIS 6 injury, and early deaths	Mixed	Adjusted mortality was lower with ICPm, but no functional outcome was reported; major caveat: administrative dataset without detailed physiological granularity.
**Alali et al. 2015 (2010–2012)**	USA	ACS TQIP registry; retrospective paediatric cohort with multilevel modelling and propensity-matched sensitivity analysis	1,705	8–9 (IQR 3–14); approximately 65% male	8–9 (IQR 3–14); approximately 65% male	Children ≤16 y with severe TBI, GCS ≤8; excluded penetrating injury, AIS 6 injury, polytrauma, and non-severe TBI	Mixed	Adjusted in-hospital mortality was lower with ICPm, but no functional outcome was reported; major caveat: administrative paediatric dataset without detailed monitoring, imaging, or physiological data.
**Al-Anazi et al. 2004 (1992–2002)**	Saudi Arabia	Single-centre retrospective cohort	178	26.4 (range 2–82); 92.7% male	26.4 (range 2–82); 92.7% male	Severe TBI: GCS ≤8 on admission; ICU admission with intubation, ventilation, sedation, and/or urgent neurosurgery; all patients had CT; no explicit exclusions beyond data availability	Intraventricular catheter / ventriculostomy	Lower mortality and better non-standardised outcome were reported with ICPm; major caveat: no adjustment and substantial risk of selection bias.

**Alkhoury et al. 2014 (2001–2006)**	USA	NTDB registry; retrospective paediatric cohort with multivariable logistic regression	3,107	8.8 ± 5.9; approximately 60–65% male	8.4 ± 6.0; approximately 60–65% male	Paediatric patients <17 y with severe blunt TBI: GCS <9, ISS >9, ICU admission, and CT evidence of TBI; excluded incomplete records and ICU LOS <24 h	Mixed	No overall mortality benefit was shown, with possible benefit limited to the GCS 3 subgroup; no functional outcome was reported; major caveat: registry-based paediatric analysis with limited physiological detail.
**Al Saiegh et al. 2020 (2000–2017)**	USA	Statewide trauma registry; retrospective observational cohort with survival analysis	36,929	41.2 ± 18.7; 76% male	46.9 ± 21.6; 73% male	Adults >18 y with severe TBI, admission GCS <9; statewide trauma registry; excluded dead on arrival; not restricted to isolated TBI; no CT-based inclusion criterion	Mixed / not specified	Log-Poisson modelling showed no mortality benefit, whereas Cox analysis suggested lower mortality with ICPm; FIM was worse in monitored patients; major caveat: discordant models with likely residual confounding.
**Biersteker et al. 2012 (Jun 2008–May 2009)**	Netherlands	POCON; prospective multicentre observational cohort	265	44 (26–54); 68% male	53 (37–69); 68% male	Severe TBI meeting BTF ICPm criteria: GCS ≤8 with abnormal CT, or GCS ≤8 with normal CT plus ≥2 of age >40 y, motor posturing, or SBP <90 mmHg; age ≥16 y; excluded admission >72 h, gunshot injury, missing CT, and non-participating-centre admission	Mixed / not specified	ICPm was not associated with lower mortality or better functional outcome after adjustment; major caveat: strong confounding by indication with worse baseline severity in monitored patients.
**Chesnut et al. 2012 (Sep 2008–Oct 2011)**	Bolivia / Ecuador	Multicentre randomised controlled trial	324	29 (22–44); 91% male	29 (22–44); 84% male	Severe TBI: GCS 3–8 on admission or deterioration within 48 h; age ≥13 y; ICU admission; excluded GCS 3 with bilateral fixed dilated pupils and unsurvivable injury	Intraparenchymal monitor; protocolised ICP-guided care versus imaging/clinical care	No mortality or functional outcome benefit was shown with ICP-guided care; major caveat: trial conducted in a resource-limited setting with a non-standard comparator pathway.
**Dawes et al. 2015 (2009–2010)**	USA	Prospective multicentre observational cohort with propensity score matching	822	42.5; approximately 75% male	42.5; approximately 75% male	Severe blunt TBI: ED GCS ≤8 and abnormal intracranial CT; prospective enrolment across trauma centres; excluded dead on arrival	Mixed / not specified	After propensity matching, inpatient mortality was lower with ICPm, but no functional outcome was reported; major caveat: observational design despite strong adjustment.
**Farahvar et al. 2012 (Jun 2000–Dec 2009)**	USA	BTF TBI-trac; prospective multicentre observational cohort	1,307	36.6 ± 19.8; 75.7% male	36.6 ± 19.8; 75.7% male	Severe TBI: GCS <9 for ≥6 h after resuscitation, arrival ≤24 h, and at risk of intracranial hypertension; included patients receiving ICP-lowering therapy in the first 2 d; excluded brain death on arrival, early non-survivable injury, and missing outcome	Mixed / not specified	Adjusted short-term mortality was lower with ICPm, but no long-term functional outcome was reported; major caveat: analysis was restricted to a treated severe TBI subgroup rather than all severe TBI.
**Foote et al. 2022 (2015–2020)**	USA	Single-centre retrospective observational cohort	123	34 (22–65); 67% male	54 (29–71); 66% male	Severe TBI defined as GCS ≤8 with abnormal CT; consecutive trauma admissions; included blunt and penetrating injury; no age restriction; excluded burns; ICPm placement at clinician discretion	Mixed: EVD, bolt, or other	No mortality benefit was shown and no validated functional outcome was reported; major caveat: small single-centre study with marked selection bias.
**Fu et al. 2020 (2012–2013)**	China	Huashan TBI database; multicentre retrospective cohort with propensity score matching	651	Approximately 50.5 ± 15.8; 75% male	Approximately 50.5 ± 15.8; 75% male	Adults >17 y with TBI, GCS ≤13, cerebral contusion >20 mL on CT, and admission ≤24 h; excluded penetrating injury, significant EDH/SDH, herniation, bilateral fixed pupils, and major mass lesions	Mixed / not specified	Functional outcome was better with ICPm in the unmatched cohort, but mortality did not differ after matching; major caveat: cohort was restricted to contusion-predominant TBI.
**Gao et al. 2013 (not stated)**	China	Retrospective operative cohort	63	47; 80% male	47; 80% male	Traumatic bifrontal contusions on CT; mixed severity; operative subgroup analysed for ICPm versus no ICPm; ICPm typically used with GCS <8, deterioration, or progressive CT change	Intraventricular catheter ± CSF drainage	No mortality difference was shown and 6-month GOS was better with ICPm; major caveat: highly selected operative subgroup.
**Griesdale et al. 2010 (May 2000–Mar 2006)**	Canada	Retrospective single-centre cohort with propensity-adjusted multivariable regression	171	35 ± 15; approximately 79% male	42 ± 18; approximately 75% male	Severe TBI: GCS ≤8 and ICU admission; excluded obeying commands within 12 h, deaths within 12 h, high cervical injury, and non-traumatic coma	EVD only	Adjusted hospital mortality was higher with EVD and no functional outcome was reported; major caveat: likely residual confounding by treatment intensity and indication.

**Haddad et al. 2011 (Feb 2001–Dec 2008)**	Saudi Arabia	Single-centre retrospective ICU cohort	477	28.0 ± 11.3; 96% male	29.3 ± 14.0; 96% male	Adults ≥18 y with severe TBI: GCS 3–8 and ICU admission; consecutive admissions; excluded brain death on admission; BTF-based ICU care, but ICPm use at clinician discretion	Intraventricular only	ICPm was not associated with lower mortality and no functional outcome was reported; major caveat: substantial confounding by clinician-directed monitor selection.
**Jitchanvichai et al. 2024 (2013–2018)**	Thailand	Single-centre retrospective cohort with propensity score matching	849	30.4 ± 18.5; 79% male	37.8 ± 19.7; 79% male	Severe TBI: post-resuscitation GCS ≤8, age ≥15 y, preoperative CT available, and level 1 trauma centre admission; excluded death before ED, no CT, intraventricular monitoring, and missing data	Intraparenchymal monitor	No mortality benefit was shown and no functional outcome was reported; major caveat: ICPm utilisation was very low, suggesting strong treatment selection.
**Kostić et al. 2011 (Feb 2008–Feb 2010)**	Serbia	Prospective quasi-randomised comparative study	61	40.97 ± 21.70; 90.6% male	43.41 ± 22.31; 82.8% male	Severe TBI: GCS ≤8 or abnormal CT with mass lesion, including midline shift >5 mm; intracranial hypertension or high risk thereof; baseline groups similar for age and GCS	Mixed: subdural, intraparenchymal, and intraventricular	No statistically significant mortality benefit was shown and no robust functional outcome difference was demonstrated; major caveat: very small underpowered quasi-randomised design.
**Lane et al. 2000 (1989, 1991–1995)**	Canada	Ontario Trauma Registry; retrospective trauma registry cohort	5,507	34.1; 66.8% male	40.7; 72.5% male	Severe TBI defined as ISS >12 and MAIS head >3 from provincial trauma registry; admitted to trauma centres; no GCS-based inclusion because GCS was incomplete and excluded from modelling	Mixed / not specified	Adjusted mortality was lower with ICPm, but no functional outcome was analysable; major caveat: GCS was excluded from modelling, with substantial residual confounding.
**Lele et al. 2019 (2012–2014)**	India	CHIRAG cohort; prospective observational cohort with IPW and multivariable regression	200	36.0 ± 13.9; 84% male	36.0 ± 13.9; 84% male	Adults ≥18 y with severe TBI: post-resuscitation GCS <8, head AIS ≥3, abnormal CT, and mechanical ventilation >48 h; included transfers and polytrauma; excluded early death <48 h; ICPm inserted ≤72 h at clinician discretion	Intraparenchymal bolt; no EVD	Adjusted in-hospital mortality was lower with ICPm, but longer-term mortality and functional outcomes were not improved; major caveat: single-centre observational design with survivor-selection constraints.
**Mauritz et al. 2008 (1998–2004)**	Austria	Registry-based prospective multicentre ICU cohort	1,856	46 (29–63); 73–74% male	53 (34–74); 73–74% male	Severe TBI: GCS <9 on admission or last pre-sedation score, AIS head >2, and ICU admission; excluded GCS ≥9, ICU LOS <4 d, and missing GCS/AIS head	Mixed / not specified	No mortality benefit was shown and no functional outcome was reported; major caveat: observational registry design with centre-level practice variation.
**Nattino et al. 2023 (Mar 2014–2019)**	Italy / Hungary	CREACTIVE cohort; prospective multicentre observational cohort with full matching propensity analysis	1,448	45.9 ± 18.5; 77.9% male	61.9 ± 20.1; 69.4% male	Adults with severe TBI meeting BTF criteria: GCS 3–8 with abnormal CT, or normal CT plus ≥2 of age >40 y, motor posturing, or SBP <90 mmHg; ICU admission ≤2 d; excluded paediatric ICU patients, ICU delay ≥3 d, pre-existing disability, palliative admission, bilateral fixed pupils, and missing 6-month outcome	Mixed / not specified	ICPm was not associated with lower mortality and was associated with worse 6-month GOSE; major caveat: residual confounding remains possible despite robust matching.
**Peled et al. 2025 (2015–2021)**	Israel	National Trauma Registry; retrospective cohort from prospectively maintained registry	2,202	Mixed age cohort; approximately 80% male	Mixed age cohort; approximately 80% male	Severe TBI: GCS 3–8 plus AIS head injury ≥3; level I trauma centre admission; excluded ED deaths and missing ICPm data	Mixed invasive ICPm	Lower 1-year mortality was reported with ICPm overall, with strongest adjusted benefit in severe injury, but no validated functional outcome was reported; major caveat: registry-based analysis with heterogeneous age strata and residual confounding.
**Robba et al. 2021 (Mar 2018–Apr 2019)**	International	SYNAPSE-ICU; prospective multicentre international observational cohort with IPTW	2,367	55 (39–69); 65% male	55 (39–69); 65% male	Adults ≥18 y with acute brain injury, including TBI, ICH, or SAH, admitted to ICU with impaired consciousness on admission or within 48 h; excluded non-ICU patients and non-acute brain injury	Mixed: parenchymal, intraventricular, or other	ICPm was associated with lower 6-month mortality only in more severe patients, with no clear benefit in less severe patients; major caveat: mixed pathology cohort rather than pure TBI.
**Schupper et al. 2019 (2010–2014)**	USA	NTDB; retrospective cohort with propensity matching	30,710	44.5 ± 18.4; 75.1% male	52.8 ± 21.1; 70.0% male	Severe blunt TBI: admission GCS 3–8; excluded non-head AIS ≥3, LOS <24 h, ED discharge, and interfacility transfers	Mixed / not specified	Higher crude mortality was seen with ICPm and no clear adjusted benefit was reported; major caveat: primary study focus was age-related monitor use rather than causal outcome estimation.

**Shafi et al. 2008 (1994–2001)**	USA	NTDB registry; retrospective multicentre cohort with multivariable logistic regression	1,646	Approximately 33 ± SEM; approximately 75% male	Approximately 33 ± SEM; approximately 75% male	Adults 20–50 y with severe blunt TBI meeting BTF criteria: GCS ≤8 with abnormal CT; ICU LOS ≥3 d; excluded death <48 h, delayed admission >24 h, AIS 3 only, and unsurvivable injury	Mixed, including ventriculostomy	Adjusted survival was worse with ICPm and functional independence was lower; major caveat: marked confounding by indication in registry data.
**Suehiro et al. 2017 (Jul 2009–Jun 2011)**	Japan	JNTDB registry; retrospective multicentre registry cohort	1,091	51.5 ± 23.5; 72.8% male	57.1 ± 24.3; 67.7% male	Severe TBI: GCS ≤8 on admission or deterioration to ≤8 within 48 h, or post-craniotomy for traumatic haematoma; registry-based inclusion using Japanese guideline framework; no strict cross-centre ICPm criteria	Mixed: intraparenchymal, subdural, intraventricular, or epidural	Lower mortality was reported with ICPm, but favourable functional outcome did not differ; major caveat: substantial treatment imbalance between groups.
**Talving et al. 2013 (Jan 2010–Dec 2011)**	USA	Prospective observational cohort with multivariable and propensity-adjusted analysis	216	40.1 ± 1.9; 79% male	48.0 ± 2.4; 70% male	Adults ≥18 y with severe blunt TBI: GCS ≤8 and head AIS ≥3, meeting BTF criteria; ICPm placed ≤24 h; excluded paediatric patients, moribund patients, and those not expected to improve	Mixed: ventriculostomy and intraparenchymal	Adjusted mortality was lower with ICPm, but no long-term functional outcome was reported; major caveat: single-centre non-randomised design.
**Yang et al. 2022 (Dec 2014–Aug 2017)**	China	CENTER-TBI China Registry; prospective multicentre observational cohort with propensity score matching	2,029	50 (37–61); approximately 78% male	50 (37–61); approximately 78% male	Severe TBI: GCS ≤8 admitted to ICU; registry-based inclusion across ICUs; excluded missing GCS, ICPm status, or discharge survival	Mixed invasive ICPm	Lower in-hospital mortality was reported with ICPm, but no validated long-term functional outcome was reported; major caveat: outcome assessment was limited to hospital-course data and discharge disposition.
**You et al. 2016 (Jan 2008–Jun 2014)**	China	Prospective single-centre observational cohort	166	74 (68–78); 42.5% male	76 (69–82); 39.5% male	Older adults ≥65 y with severe TBI: GCS <9, abnormal CT, and admission ≤24 h; ICPm indication based on severe TBI plus abnormal CT, or normal CT with ≥2 of age >40 y, motor posturing, or SBP <90 mmHg; excluded death <24 h, brain death on admission, and delayed presentation >24 h	Intraventricular ICPm	ICPm was associated with lower in-hospital mortality and better 6-month GOS; major caveat: small single-centre non-randomised elderly cohort.
**Zeng et al. 2013 (Jan 2010–Jan 2012)**	China	Prospective controlled observational study	168	42.5; approximately 61% male	43; approximately 67% male	Patients aged 15–70 y with moderate–severe TBI, GCS <13, and normal baseline renal function; excluded pre-existing kidney injury, abdominal injury, and lung contusion; ICPm use based on BTF criteria but not strictly enforced	Intraventricular ICPm with CSF drainage	Functional outcome at 6 months was better with ICPm and mortality trended lower; major caveat: small non-randomised single-centre study.

NB: Values in the n column refer to the comparative ICP-monitored versus non-ICP-monitored sample included in this review, where extractable. Age is reported as mean ± standard deviation or median (interquartile range) according to the original publication. Where subgroup data were available, values are presented separately for ICP-monitored and non-ICP-monitored groups. Population descriptors, monitor type, and inclusion criteria are summarized as reported in the original studies. “Mixed” indicates that multiple invasive ICP monitoring modalities were grouped, or that the specific device type was not consistently reported.

Abbreviations: AIS, Abbreviated Injury Scale; BTF, Brain Trauma Foundation; CSF, cerebrospinal fluid; CT, computed tomography; ED, emergency department; EDH, epidural haematoma; EVD, external ventricular drain; GCS, Glasgow Coma Scale; GOSE, Glasgow Outcome Scale–Extended; ICH, intracerebral haemorrhage; ICPm, intracranial pressure monitoring; ICU, intensive care unit; IP, intraparenchymal; IPTW, inverse probability of treatment weighting; ISS, Injury Severity Score; IV, intraventricular; LOS, length of stay; MAIS, maximum Abbreviated Injury Scale; MV, mechanical ventilation; NS, not specified; PSM, propensity score matching; SAH, subarachnoid haemorrhage; SBP, systolic blood pressure; SDH, subdural haematoma; SEM, standard error of the mean; TBI, traumatic brain injury; TQIP, Trauma Quality Improvement Program.

SDC 6b. GOS and GOS-E/GOSE outcomes

Study & Year	Timepoint	Outcome measure	GOS / GOS-E / GOSE		Reported effect / p value	Notes
			ICPm	No ICPm		
Al-Anazi et al. 2004	Discharge / in-hospital outcome	GOS	3: 6 (12%)<br>4: 13 (25%)<br>5: 21 (40%)	3: 2 (2%)<br>4: 10 (8%)<br>5: 26 (21%)	p<0.001	Satisfactory outcome defined by study as GOS 3–5.
Biersteker et al. 2012	6 months	GOSE	2: 2 (3.6%)<br>3: 14 (25%)<br>4: 6 (11%)<br>5: 12 (21%)<br>6: 10 (18%)<br>7: 4 (7.0%); 8: 2 (3.6%)	2: 0 (0%)<br>3: 9 (11%)<br>4: 6 (7.5%)<br>5: 11 (14%)<br>6: 13 (16%)<br>7: 18 (23%)<br>8: 18 (23%)	Unfavorable survival GOSE 2–4: 22 (44%) vs 15 (20%), p<0.01	Reported among survivors.
Chesnut et al. 2012	6 months	GOS-E	1: 56 (39%)<br>2–4: 24 (17%)<br>5–8: 63 (44%)	1: 67 (44%)<br>2–4: 26 (17%)<br>5–8: 60 (39%)	Proportional OR 1.23 [0.77–1.96], p=0.40	Composite functional/cognitive percentile score: median 56 (IQR 22–77) vs 53 (IQR 21–76); proportional OR 1.09 [0.74–1.58], p=0.49.
Fu et al. 2020<br>Gao et al. 2013<br>Lele et al. 2019	6 months	GOS	3: 33 (9.6%)<br>4: 127 (37.1%)<br>5: 109 (31.9%)	3: 28 (15.7%)<br>4: 42 (23.6%)<br>5: 48 (27.0%)	p<0.001	Unmatched cohort; after propensity score matching, unfavorable outcome OR 0.86 [0.59–1.25], p=0.433.
	6 months	GOS	4.21 ± 0.88	3.32 ± 1.20	p=0.025	Operative subgroup; mean GOS.
	3 months	GOSE	Median 5 (IQR 3–7)	Median 5 (IQR 1–7)	p=0.31	No significant difference.
Lele et al. 2019	6 months	GOSE	Median 5 (IQR 3–7)	Median 5 (IQR 3–7)	p=0.53	Adjusted aRR for 6-month unfavorable outcome 0.84 [0.53–1.35], p=0.479.
Lele et al. 2019	12 months	GOSE	Median 7 (IQR 4–8)	Median 6 (IQR 4–8)	p=0.45	No significant difference.
Nattino et al. 2023	6 months	GOSE	1–2: 197 (39.2%)<br>3: 125 (24.9%)<br>4: 42 (8.3%)<br>5: 39 (7.8%)<br>6: 40 (8.0%)<br>7: 29 (5.8%)<br>8: 31 (6.2%)	1–2: 384 (40.6%)<br>3: 145 (15.3%)<br>4: 64 (6.8%)<br>5: 64 (6.8%)<br>6: 70 (7.4%)<br>7: 67 (7.1%)<br>8: 61 (6.5%)	p=0.005	Overall GOSE distribution was significantly worse in the ICPm group.
Robba et al. 2021	6 months	GOSE <5 subgroup analysis	NR	NR	Both pupils reactive subgroup: OR 1.34 [1.11–1.63]; ≥1 unreactive pupil subgroup: OR 0.38 [0.26–0.56]	No extractable overall group-level GOSE distribution was reported for ICPm versus no ICPm. Adjusted subgroup analyses were reported for unfavorable neurological outcome, defined as GOSE <5.
You et al. 2016	6 months	GOS	3.0 ± 1.4	2.5 ± 1.2	p=0.014	Mean GOS.
Zeng et al. 2013	6 months	GOS	1: 2 (2.6%)<br>2: 3 (3.9%)<br>3: 19 (24.7%)<br>4: 18 (23.4%)<br>5: 35 (45.5%)	1: 10 (11.0%)<br>2: 6 (6.6%)<br>3: 31 (34.1%)<br>4: 25 (27.5%)<br>5: 19 (20.9%)	p<0.01	Overall GOS distribution was significantly better in the ICPm group.

NB: GOS and GOS-E/GOSE outcomes are reported as defined in the original publication. Timepoints are reported as stated in the original study. For GOS and GOS-E/GOSE category distributions, scale numbers are used in place of verbal labels to improve table concision. GOS: 1 = death, 2 = vegetative state, 3 = severe disability, 4 = moderate disability, 5 = good recovery. GOS-E/GOSE: 1 = death, 2 = vegetative state, 3 = lower severe disability, 4 = upper severe disability, 5 = lower moderate disability, 6 = upper moderate disability, 7 = lower good recovery, 8 = upper good recovery. NR indicates not reported.

Abbreviations: aRR, adjusted relative risk; GOS, Glasgow Outcome Scale; GOS-E/GOSE, Glasgow Outcome Scale–Extended; ICPm, intracranial pressure monitoring; IQR, interquartile range; NR, not reported; OR, odds ratio; SD, standard deviation.

Unreported: Agrawal et al. 2015; Aiolfi et al. 2017; Alali et al. 2013; Alali et al. 2015; Alkhoury et al. 2014; Al Saiegh et al. 2020; Dawes et al. 2015; Farahvar et al. 2012; Foote et al. 2022; Griesdale et al. 2010; Haddad et al. 2011; Jitchanvichai et al. 2024; Kostić et al. 2011; Lane et al. 2000; Mauritz et al. 2008; Peled et al. 2025; Schupper et al. 2019; Shafi et al. 2008; Suehiro et al. 2017; Talving et al. 2013; Yang et al. 2022. Discharge disposition only: Foote et al. 2022; Peled et al. 2025.

SDC 6c. Functional outcomes in ICP-monitored versus non-ICP-monitored groups (Descriptive, per study)

Study & Year	Outcome measure	Timepoint	ICPm Group	No ICPm Group	Reported effect / p value	Notes
**Chesnut et al. 2012**	Composite functional/cognitive percentile score	6 months	Median 56 (IQR 22–77)	Median 53 (IQR 21–76)	Proportional OR 1.09 [0.74–1.58], p=0.49	Primary outcome; higher score indicates better outcome
	GOS-E	6 months	Death 56 (39%); unfavorable 24 (17%); favorable 63 (44%)	Death 67 (44%); unfavorable 26 (17%); favorable 60 (39%)	Proportional OR 1.23 [0.77–1.96], p=0.40	Higher value favors pressure-monitoring group
**Biersteker et al. 2012**	GOSE distribution in survivors	6 months	Vegetative state 2 (3.6%); lower severe disability 14 (25%); upper severe disability 6 (11%); lower moderate disability 12 (21%); upper moderate disability 10 (18%); lower good recovery 4 (7.0%); upper good recovery 2 (3.6%); unknown 7 (12%)	Vegetative state 0 (0%); lower severe disability 9 (11%); upper severe disability 6 (7.5%); lower moderate disability 11 (14%); upper moderate disability 13 (16%); lower good recovery 18 (23%); upper good recovery 18 (23%); unknown 5 (6.3%)	-	Reported among survivors
	Unfavorable outcome (GOSE 1–4)	6 months	81 (74%)	67 (53%)	p=0.001	Includes death
	Unfavorable survival (GOSE 2–4)	6 months	22 (44%)	15 (20%)	p<0.01	Restricted to survivors
**You et al. 2016**	GOS	6 months	3.0 ± 1.4	2.5 ± 1.2	p=0.014	Higher mean GOS in ICP-monitored group
**Lele et al. 2019**	GOSE	3 months	Median 5 (IQR 3–7)	Median 5 (IQR 1–7)	p=0.31	No significant difference
	GOSE	6 months	Median 5 (IQR 3–7)	Median 5 (IQR 3–7)	p=0.53	No significant difference
	GOSE	12 months	Median 7 (IQR 4–8)	Median 6 (IQR 4–8)	p=0.45	No significant difference
	Unfavorable functional outcome (GOSE 1–4)	6 months	50/126 (39.7%)	31/74 (41.9%)	aRR 0.84 [0.53–1.35], p=0.479	Adjusted model showed no significant association
**Nattino et al. 2023**	GOSE distribution	6 months	Death/vegetative state 197 (39.2%); lower severe disability 125 (24.9%); upper severe disability 42 (8.3%); lower moderate disability 39 (7.8%); upper moderate disability 40 (8.0%); lower good recovery 29 (5.8%); upper good recovery 31 (6.2%)	Death/vegetative state 384 (40.6%); lower severe disability 145 (15.3%); upper severe disability 64 (6.8%); lower moderate disability 64 (6.8%); upper moderate disability 70 (7.4%); lower good recovery 67 (7.1%); upper good recovery 61 (6.5%)	p=0.005	Matched/weighted analysis; overall GOSE distribution significantly worse in ICPm group
	Unfavorable functional outcome (GOSE 1–4)	6 months	403/503 (80.1%)	657/945 (69.5%)	-	Derived from reported GOSE category distribution
	Favorable functional outcome (GOSE 5–8)	6 months	100/503 (19.9%)	288/945 (30.5%)	-	Derived from reported GOSE category distribution
**Fu et al. 2020**	GOS distribution	6 months	Severe disability 33 (9.6%); moderate disability 127 (37.1%); good recovery 109 (31.9%)	Severe disability 28 (15.7%); moderate disability 42 (23.6%); good recovery 48 (27.0%)	p<0.001	Reported among survivors/categories shown in paper
	Unfavorable functional outcome (GOS 1–3)	6 months after PSM	79/237 (33.3%)	87/237 (36.7%)	OR 0.86 [0.59–1.25], p=0.433	PSM cohort; no significant difference
	Unfavorable functional outcome (GOS 1–3)	6 months	106/342 (31.0%)	130/178 (73.0%)	OR 0.31 [0.22–0.43], p<0.001	Unmatched cohort

	Favorable functional outcome (GOS 4–5)	6 months	236/342 (69.0%)	48/178 (27.0%)	OR 3.22 [2.31–4.50], p<0.001	Unmatched cohort; inverse framing of unfavorable outcome
**Foote et al. 2022**	Discharge destination: home	Discharge	23/58 (39.7%)	15/65 (23.1%)	p=0.06	Trend toward more home discharge in ICPm group
	Discharge destination: skilled nursing / long-term acute care	Discharge	15/58 (25.9%)	27/65 (41.5%)	p=0.08	Trend toward fewer skilled nursing / LTAC discharges in ICPm group
	Discharge destination: rehabilitation	Discharge	0/58 (0%)	1/65 (1.5%)	p>0.99	Rare event
	Discharge destination: hospice	Discharge	0/58 (0%)	3/65 (4.6%)	p=0.25	Rare event
**Robba et al. 2021**	Unfavorable neurological outcome (GOSE <5)	6 months	NR	NR	OR 1.34 [1.11–1.63]	Both pupils reactive subgroup; adjusted analysis
	Unfavorable neurological outcome (GOSE <5)	6 months	NR	NR	OR 0.38 [0.26–0.56]	At least one unreactive pupil subgroup; adjusted analysis
**Al-Anazi et al. 2004**	Glasgow Outcome Scale category distribution	Discharge / in-hospital outcome	Good recovery 21 (40%); moderate disability 13 (25%); severe disability 6 (12%)	Good recovery 26 (21%); moderate disability 10 (8%); severe disability 2 (2%)	-	Category counts as reported
	Satisfactory outcome	Discharge / in-hospital outcome	40/52 (76.9%)	48/126 (38.1%)	p<0.001	Defined by study as good recovery + moderate disability + severe disability
	Poor outcome	Discharge / in-hospital outcome	12/52 (23.1%)	78/126 (61.9%)	p<0.001	Defined by study as vegetative state + death
**Zeng et al. 2013**	GOS distribution	6 months	Death 2 (2.6%); vegetative state 3 (3.9%); severe disability 19 (24.7%); moderate disability 18 (23.4%); good recovery 35 (45.5%)	Death 10 (11.0%); vegetative state 6 (6.6%); severe disability 31 (34.1%); moderate disability 25 (27.5%); good recovery 19 (20.9%)	p<0.01	Overall GOS distribution significantly better in ICPm group
	Favorable functional outcome (GOS 4–5)	6 months	53/77 (68.8%)	44/91 (48.4%)	-	Derived from reported GOS distribution
	Unfavorable functional outcome (GOS 1–3)	6 months	24/77 (31.2%)	47/91 (51.6%)	-	Derived from reported GOS distribution
**Gao et al. 2013**	GOS	6 months	4.21 ± 0.88	3.32 ± 1.20	p=0.025	Higher mean GOS in ICPm group
	Favorable functional outcome (GOS 4–5)	6 months	31/39 (79.5%)	11/24 (45.8%)	-	Derived from reported mean/individual outcome distribution in operative subgroup
	Unfavorable functional outcome (GOS 1–3)	6 months	8/39 (20.5%)	13/24 (54.2%)	-	Derived from reported operative subgroup outcomes
**Agrawal et al. 2015**	Poor functional outcome (GOS ≤3)	6 months	PS-adjusted probability 0.213	PS-adjusted probability 0.147	p=0.168	Among 6-month survivors with available follow-up; no significant effect of ICPM
	Poor functional outcome (GOS ≤3)	6 months, excluding early decompressive craniectomy	PS-adjusted probability 0.179	PS-adjusted probability 0.105	p=0.076	Subgroup excluding eDC; no significant effect of ICPM
**Shafi et al. 2008**	FIM at discharge	Discharge	5.9 ± 0.16	7.9 ± 0.14	p<0.001	Lower functional independence in ICPm group
	Poor functional outcome	Discharge	Reported as worse functional outcome in ICPm group	Reported as better functional outcome in no-ICPm group	Adjusted OR for poor functional outcome 1.71 [1.30–2.25], p<0.001	Derived from multivariable model
**Aiolfi et al. 2017**	Functional independence measure (good)	Discharge	169/1519 (16.6%)	2212/7440 (29.7%)	p<0.001	Among survivors only
	Poor functional independence	Discharge	Reported as worse in ICPm group	Reported as better in no-ICPm group	OR 1.889 [1.575–2.264], p<0.001	Adjusted analysis
**Al Saiegh et al. 2020**	FIM at discharge	Discharge	9.53 ± 5.07	16.21 ± 4.91	p<0.0001	Higher mean FIM in no-ICPm group; among patients alive at discharge
	FIM at discharge	Discharge	NR	NR	Adjusted beta -5.02 [95% CI -5.22 to -4.82], p<0.0001	Multivariate linear regression; ICP monitor placement independently associated with lower FIM

**Suehiro et al. 2017**	Favorable functional outcome	Discharge	89/305 (29.2%)	236/786 (30.0%)	p=0.80	Defined by study as good recovery or moderate disability
	Unfavorable functional outcome	Discharge	216/305 (70.8%)	550/786 (70.0%)	-	Derived from reported favorable outcome counts
**Peled et al. 2025**	Discharge to rehabilitation	Discharge	Reported as more frequent in ICPm group	Reported as less frequent in no-ICPm group	p<0.001	Disposition outcome; not a validated functional scale

NB: Functional outcomes are reported as defined in the original publication and may include composite functional/cognitive scores, GOS, GOS-E/GOSE, FIM, favorable or unfavorable outcome, or discharge disposition where explicitly presented by study group. Timepoints are reported as stated in the original study. Derived favorable/unfavorable outcome categories were calculated only when the full underlying outcome distribution was reported. “NR” indicates that no extractable validated functional outcome was reported for ICP-monitored versus non-ICP-monitored groups.
Abbreviations: ICPm, intracranial pressure monitoring; GOS, Glasgow Outcome Scale; GOS-E/GOSE, Glasgow Outcome Scale–Extended; FIM, Functional Independence Measure; IQR, interquartile range; SD, standard deviation; SEM, standard error of the mean; OR, odds ratio; aRR, adjusted relative risk; NR, not reported.
Unreported: Lane et al. 2000; Farahvar et al. 2012; Kostić et al. 2011; Talving et al. 2013; Dawes et al. 2015; Jitchanvichai et al. 2024; Haddad et al. 2011; Griesdale et al. 2010; Mauritz et al. 2008; Alali et al. 2013; Alali et al. 2015; Schupper et al. 2019; Alkhoury et al. 2014; Yang et al. 2022.

SDC 6d. Functional outcomes in ICPm vs non-ICPm groups (Per outcome measure)

Study & Year	Timepoint	GOS		GOS-E / GOSE		FIM		Favorable outcome		Unfavorable outcome		Other functional outcome / Notes
		ICPm	No ICPm	ICPm	No ICPm	ICPm	No ICPm	ICPm	No ICPm	ICPm	No ICPm	
Chesnut et al. 2012	6 months	-	-	1: 56 (39%)<br>2–4: 24 (17%)<br>5–8: 63 (44%)	1: 67 (44%)<br>2–4: 26 (17%)<br>5–8: 60 (39%)	-	-	63 (44%)	60 (39%)	24 (17%)	26 (17%)	Composite functional/cognitive percentile score: median 56 (IQR 22–77) vs 53 (IQR 21–76); proportional OR 1.09 [0.74–1.58], p=0.49. GOS-E proportional OR 1.23 [0.77–1.96], p=0.40
Biersteker et al. 2012	6 months	-	-	2: 2 (3.6%)<br>3: 14 (25%)<br>4: 6 (11%)<br>5: 12 (21%)<br>6: 10 (18%)<br>7: 4 (7.0%)<br>8: 2 (3.6%)	2: 0<br>3: 9 (11%)<br>4: 6 (7.5%)<br>5: 11 (14%)<br>6: 13 (16%)<br>7: 18 (23%)<br>8: 18 (23%)	-	-	-	-	81 (74%)	67 (53%)	Reported among survivors; unfavorable survival (GOSE 2–4): 22 (44%) vs 15 (20%), p<0.01
You et al. 2016	6 months	3.0 ± 1.4	2.5 ± 1.2	-	-	-	-	-	-	-	-	p=0.014
Lele et al. 2019	3 months	-	-	Median 5 (IQR 3–7)	Median 5 (IQR 1–7)	-	-	-	-	-	-	p=0.31
	6 months	-	-	Median 5 (IQR 3–7)	Median 5 (IQR 3–7)	-	-	-	-	50/126 (39.7%)	31/74 (41.9%)	Adjusted aRR for 6-month unfavorable outcome 0.84 [0.53–1.35], p=0.479
	12 months	-	-	Median 7 (IQR 4–8)	Median 6 (IQR 4–8)	-	-	-	-	-	-	p=0.45
Nattino et al. 2023	6 months	-	-	1–2: 197 (39.2%)<br>3: 125 (24.9%)<br>4: 42 (8.3%)<br>5: 39 (7.8%)<br>6: 40 (8.0%)<br>7: 29 (5.8%)<br>8: 31 (6.2%)	1–2: 384 (40.6%)<br>3: 145 (15.3%)<br>4: 64 (6.8%)<br>5: 64 (6.8%)<br>6: 70 (7.4%)<br>7: 67 (7.1%)<br>8: 61 (6.5%)	-	-	100/503 (19.9%)	288/945 (30.5%)	403/503 (80.1%)	657/945 (69.5%)	Overall GOSE distribution significantly worse in ICPm group, p=0.005
Fu et al. 2020	6 months	3: 33 (9.6%)<br>4: 127 (37.1%)<br>5: 109 (31.9%)	3: 28 (15.7%)<br>4: 42 (23.6%)<br>5: 48 (27.0%)	-	-	-	-	236/342 (69.0%)	48/178 (27.0%)	106/342 (31.0%)	130/178 (73.0%)	Unmatched cohort. After PSM, unfavorable outcome 79/237 (33.3%) vs 87/237 (36.7%), OR 0.86 [0.59–1.25], p=0.433
Robba et al. 2021	6 months	-	-	GOSE <5 analyzed in	GOSE <5 analyzed in	-	-	-	-	-	-	No clean overall raw GOSE table extractable. Adjusted subgroup

				subgroups only	subgroups only							analyses: unfavorable neurological outcome (GOSE <5) OR 1.34 [1.11–1.63] in both pupils reactive subgroup; OR 0.38 [0.26–0.56] in ≥1 unreactive pupil subgroup
**Al-Anazi et al. 2004**	Discharge / in-hospital outcome	3: 6 (12%)<br>4: 13 (25%)<br>5: 21 (40%)	3: 2 (2%)<br>4: 10 (8%)<br>5: 26 (21%)	-	-	-	-	40/52 (76.9%)	48/126 (38.1%)	12/52 (23.1%)	78/126 (61.9%)	Satisfactory outcome defined by study as GOS 3–5; poor outcome as vegetative state + death; p<0.001
**Zeng et al. 2013**	6 months	1: 2 (2.6%)<br>2: 3 (3.9%)<br>3: 19 (24.7%)<br>4: 18 (23.4%)<br>5: 35 (45.5%)	1: 10 (11.0%)<br>2: 6 (6.6%)<br>3: 31 (34.1%)<br>4: 25 (27.5%)<br>5: 19 (20.9%)	-	-	-	-	53/77 (68.8%)	44/91 (48.4%)	24/77 (31.2%)	47/91 (51.6%)	Overall GOS distribution significantly better in ICPm group, p<0.01
**Gao et al. 2013**	6 months	4.21 ± 0.88	3.32 ± 1.20	-	-	-	-	31/39 (79.5%)	11/24 (45.8%)	8/39 (20.5%)	13/24 (54.2%)	p=0.025; operative subgroup
**Agrawal et al. 2015**	6 months	-	-	-	-	-	-	-	-	-	-	No raw functional outcome counts extractable. PS-adjusted probability of poor outcome (GOS ≤3): 0.213 vs 0.147, p=0.168. Excluding early decompressive craniectomy: 0.179 vs 0.105, p=0.076
**Shafi et al. 2008**	Discharge	-	-	-	-	5.9 ± 0.16	7.9 ± 0.14	-	-	Adjusted OR poor outcome = 1.71 [1.30-2.25], p<0.001	-	Lower functional independence in ICPm group
**Aiolfi et al. 2017**	Discharge	-	-	-	-	169/1519 (16.6%)	2212/7440 (29.7%)	-	-	-	-	Among survivors only; poor functional independence OR 1.889 [1.575–2.264], p<0.001
**Al Saiegh et al. 2020**	Discharge	-	-	-	-	9.53 ± 5.07	16.21 ± 4.91	-	-	-	-	Multivariate beta for ICP monitor placement and lower FIM: -5.02 [95% CI -5.22 to -4.82], p<0.0001;

												among survivors at discharge
**Suehiro et al. 2017**	Discharge	-	-	-	-	-	-	89/305 (29.2%)	236/786 (30.0%)	216/305 (70.8%)	550/786 (70.0%)	Favorable outcome defined by study as good recovery or moderate disability; p=0.80

NB: Functional outcomes are reported as defined in the original publication and may include composite functional/cognitive scores, GOS, GOS-E/GOSE, FIM, or favorable/unfavorable outcome. Timepoints are reported as stated in the original study. Derived favorable or unfavorable outcome categories were calculated only when the full underlying outcome distribution was reported. Studies without an extractable validated functional outcome for ICP-monitored versus non-ICP-monitored groups were omitted from the table body and are listed below. Foote et al. 2022 and Peled et al. 2025 reported discharge disposition only and were therefore not included in the main functional outcome table.
Abbreviations: ICPm, intracranial pressure monitoring; GOS, Glasgow Outcome Scale; GOS-E/GOSE, Glasgow Outcome Scale–Extended; FIM, Functional Independence Measure; IQR, interquartile range; SD, standard deviation; OR, odds ratio; aRR, adjusted relative risk; NR, not reported.
Unreported: Lane et al. 2000; Farahvar et al. 2012; Kostić et al. 2011; Talving et al. 2013; Dawes et al. 2015; Jitchanvichai et al. 2024; Haddad et al. 2011; Griesdale et al. 2010; Mauritz et al. 2008; Alali et al. 2013; Alali et al. 2015; Schupper et al. 2019; Alkhoury et al. 2014; Yang et al. 2022. Discharge disposition only: Foote et al. 2022; Peled et al. 2025.

SDC 6e. FIM outcomes

Study & Year	Timepoint	Outcome measure	FIM / Functional independence		Reported effect / p value	Notes
			ICPm	No ICPm		
Shafi et al. 2008	Discharge	FIM	5.9 ± 0.16	7.9 ± 0.14	Adjusted OR for poor functional outcome 1.71 [1.30–2.25], p<0.001	Lower functional independence in ICPm group
Al Saiegh et al. 2020	Discharge	FIM	9.53 ± 5.07	16.21 ± 4.91	Multivariate beta -5.02 [95% CI -5.22 to -4.82], p<0.0001	Among patients alive at discharge

NB: FIM outcomes are reported as defined in the original publication and include only studies reporting group-level FIM scores. Timepoints are reported as stated in the original study. Al Saiegh et al. reported FIM among patients alive at discharge.
Abbreviations: ICPm, intracranial pressure monitoring; FIM, Functional Independence Measure; OR, odds ratio.
Unreported: Lane et al. 2000; Farahvar et al. 2012; Kostić et al. 2011; Biersteker et al. 2012; Talving et al. 2013; Dawes et al. 2015; You et al. 2016; Lele et al. 2019; Nattino et al. 2023; Fu et al. 2020; Foote et al. 2022; Robba et al. 2021; Jitchanvichai et al. 2024; Al-Anazi et al. 2004; Haddad et al. 2011; Zeng et al. 2013; Gao et al. 2013; Griesdale et al. 2010; Agrawal et al. 2015; Mauritz et al. 2008; Aiolfi et al. 2017; Alali et al. 2013; Alali et al. 2015; Schupper et al. 2019; Alkhoury et al. 2014; Suehiro et al. 2017; Yang et al. 2022; Peled et al. 2025.

SDC 6f. Favorable vs unfavorable functional outcomes

Study & Year	Timepoint	Favorable outcome		Unfavorable outcome		Reported effect / p value	Notes
		ICPm	No ICPm	ICPm	No ICPm		
**Chesnut et al. 2012**	6 months	63 (44%)	60 (39%)	24 (17%)	26 (17%)	Proportional OR 1.23 [0.77–1.96], p=0.40	GOS-E; favorable and unfavorable categories as reported
**Biersteker et al. 2012**	6 months	-	-	81 (74%)	67 (53%)	-	Unfavorable outcome includes death (GOSE 1–4); among survivors, unfavorable survival (GOSE 2–4): 22 (44%) vs 15 (20%), p<0.01
**Lele et al. 2019**	6 months	-	-	50/126 (39.7%)	31/74 (41.9%)	aRR 0.84 [0.53–1.35], p=0.479	Unfavorable outcome defined as GOSE 1–4
**Nattino et al. 2023**	6 months	100/503 (19.9%)	288/945 (30.5%)	403/503 (80.1%)	657/945 (69.5%)	p=0.005	Favorable = GOSE 5–8; unfavorable = GOSE 1–4
**Fu et al. 2020**	6 months	236/342 (69.0%)	48/178 (27.0%)	106/342 (31.0%)	130/178 (73.0%)	OR 0.31 [0.22–0.43], p<0.001	Unmatched cohort; after PSM, unfavorable outcome 79/237 (33.3%) vs 87/237 (36.7%), OR 0.86 [0.59–1.25], p=0.433
**Al-Anazi et al. 2004**	Discharge / in-hospital outcome	40/52 (76.9%)	48/126 (38.1%)	12/52 (23.1%)	78/126 (61.9%)	p<0.001	Favorable defined by study as satisfactory outcome (GOS 3–5); unfavorable as poor outcome (vegetative state + death)
**Zeng et al. 2013**	6 months	53/77 (68.8%)	44/91 (48.4%)	24/77 (31.2%)	47/91 (51.6%)	p<0.01	Favorable = GOS 4–5; unfavorable = GOS 1–3
**Gao et al. 2013**	6 months	31/39 (79.5%)	11/24 (45.8%)	8/39 (20.5%)	13/24 (54.2%)	p=0.025	Derived from operative subgroup 6-month GOS
**Aiolfi et al. 2017**	Discharge	169/1519 (16.6%)	2212/7440 (29.7%)	-	-	Poor functional independence OR 1.889 [1.575–2.264], p<0.001	Favorable outcome represented by good functional independence; among survivors only
**Suehiro et al. 2017**	Discharge	89/305 (29.2%)	236/786 (30.0%)	216/305 (70.8%)	550/786 (70.0%)	p=0.80	Favorable defined by study as good recovery or moderate disability

NB: Favorable and unfavorable outcomes are reported as defined in the original publication. Timepoints are reported as stated in the original study. Derived favorable or unfavorable categories were calculated only when the full underlying outcome distribution was reported. Where only one dichotomous functional outcome was explicitly reported, the complementary category was derived from the study totals when appropriate. Aiolfi et al. 2017 reported good functional independence and adjusted poor functional independence rather than GOS/GOSE-based favorable or unfavorable outcome. Foote et al. 2022 and Peled et al. 2025 reported discharge disposition only and were not included in this table.
Abbreviations: ICPm, intracranial pressure monitoring; GOS, Glasgow Outcome Scale; GOS-E/GOSE, Glasgow Outcome Scale–Extended; OR, odds ratio; aRR, adjusted relative risk.
Unreported: Lane et al. 2000; Farahvar et al. 2012; Kostić et al. 2011; Talving et al. 2013; Dawes et al. 2015; You et al. 2016; Robba et al. 2021; Jitchanvichai et al. 2024; Haddad et al. 2011; Griesdale et al. 2010; Agrawal et al. 2015; Mauritz et al. 2008; Alali et al. 2013; Alali et al. 2015; Al Saiegh et al. 2020; Schupper et al. 2019; Alkhoury et al. 2014; Yang et al. 2022. Discharge disposition only: Foote et al. 2022; Peled et al. 2025.

SDC 6g - Complications and treatment burden in ICP-monitored versus non-ICP-monitored groups

Study & Year	Variable	ICPm Group	No ICPm Group	Reported effect / p value	Notes
Chesnut et al. 2012	ICP catheter-related adverse events	10 (6%)	-	-	Included catheter malfunction 4 (3%), unplanned removal 4 (3%), hemorrhage 2 (1%)
	Any serious adverse event	70 (45%)	76 (46%)	p=0.91	No significant difference
	Infections	13 (8%)	10 (6%)	p=0.52	Serious adverse events category
	Nervous system adverse events (excluding infection)	19 (12%)	29 (17%)	p=0.21	Serious adverse events category
	Respiratory adverse events (excluding infection)	9 (6%)	8 (5%)	p=0.81	Serious adverse events category
	Cardiovascular adverse events	17 (11%)	13 (8%)	p=0.44	Serious adverse events category
	Decubitus ulcers	19 (12%)	8 (5%)	p=0.03	More frequent in pressure-monitoring group
You et al. 2016	Infection	3/80 (3.8%)	0/86 (0%)	-	Complication related to ICP monitoring reported only in ICPm group
	Hemorrhage	7/80 (8.7%)	0/86 (0%)	-	Complication related to ICP monitoring reported only in ICPm group
Nattino et al. 2023	Respiratory complications and infections	Higher in ICPm group	Not separately quantified	p<0.05	After weighting, respiratory complications and infections were significantly more common in monitored patients; other complications were similar.
Fu et al. 2020	Intracranial infection	11/342 (3.2%)	8/309 (2.6%)	p=0.633	No significant difference
	Hydrocephalus	3/342 (0.9%)	10/309 (3.2%)	p=0.026	More frequent in no-ICPm group
	CSF leak	2/342 (0.6%)	11/309 (3.6%)	p=0.004	More frequent in no-ICPm group
Foote et al. 2022	Pneumonia	16/58 (27.6%)	2/65 (3.1%)	p<0.001	More frequent in ICPm group
Robba et al. 2021	Catheter misposition	46 (13%)	-	-	Reported only in monitored patients
	Accidental catheter removal	31 (9%)	-	-	Reported only in monitored patients
	Faulty or broken catheter	41 (11%)	-	-	Reported only in monitored patients
	Site infection	8 (2%)	-	-	Reported only in monitored patients
Al-Anazi et al. 2004	Catheter obstruction	2/52 (3.8%)	-	-	Reported only in the ICPm group; no parallel no-ICPm comparison
Zeng et al. 2013	Acute kidney injury	5/77 (6.5%)	12/91 (13.2%)	p=0.047	More frequent in no-ICPm group
Shafi et al. 2008	Pneumonia	262/708 (37.0%)	216/938 (23.0%)	p<0.001	More frequent in ICPm group

	Infection	276/708 (39.0%)	225/938 (24.0%)	p<0.001	More frequent in ICPm group
	Renal failure	19/708 (2.7%)	10/938 (1.1%)	p=0.01	More frequent in ICPm group
Aiolfi et al. 2017	Infection	2238/9898 (22.6%)	1519/6671 (22.8%)	OR 2.282 [2.015–2.584], p<0.001	Adjusted analysis reported higher infectious complications in ICPm group despite similar crude proportions.
	Sepsis	193/9898 (2.0%)	88/6671 (1.3%)	p<0.001	More frequent in ICPm group

NB: Complications are reported as defined in the original publication and include only studies with extractable medical or device-related complication data by ICP-monitored versus non-ICP-monitored groups. Device-specific complications reported only within the ICP-monitored group are included where no parallel comparison group exists. Studies reporting treatment burden, interventions, monitoring findings, or resource-use variables without true complication outcomes were not included in the main complications table.

Abbreviations: ICPm, intracranial pressure monitoring; CSF, cerebrospinal fluid; OR, odds ratio.

Unreported: Lane et al. 2000; Farahvar et al. 2012; Kostić et al. 2011; Biersteker et al. 2012; Talving et al. 2013; Dawes et al. 2015; Lele et al. 2019; Jitchanvichai et al. 2024; Haddad et al. 2011; Gao et al. 2013; Griesdale et al. 2010; Agrawal et al. 2015; Mauritz et al. 2008; Alali et al. 2013; Alali et al. 2015; Al Saiegh et al. 2020; Schupper et al. 2019; Alkhoury et al. 2014; Suehiro et al. 2017; Yang et al. 2022; Peled et al. 2025.
