# Supplementary material for: Impacts of intracranial pressure monitoring on mortality and length of stay in severe traumatic brain injury: a systematic review and meta-analysis
Source: Eur J Trauma Emerg Surg. 2026 Aug 12;52(1):249. doi: 10.1007/s00068-026-03302-5 (PMC13468859; doi:10.1007/s00068-026-03302-5)
Supplement: Supplementary file 6 [file 68_2026_3302_MOESM6_ESM.pdf]

Supplementary Digital Content 1. PRISMA 2020 Checklist

Section and Topic	Item #	Checklist item	Location where item is reported
TITLE			
Title	1	Identify the report as a systematic review.	Title page; Abstract
ABSTRACT			
Abstract	2	See the PRISMA 2020 for Abstracts checklist.	Abstract
INTRODUCTION			
Rationale	3	Describe the rationale for the review in the context of existing knowledge.	Background
Objectives	4	Provide an explicit statement of the objective(s) or question(s) the review addresses.	Background
METHODS			
Eligibility criteria	5	Specify the inclusion and exclusion criteria for the review and how studies were grouped for the syntheses.	Methods: Inclusion criteria; Synthesis and analysis
Information sources	6	Specify all databases, registers, websites, organisations, reference lists and other sources searched or consulted to identify studies. Specify the date when each source was last searched or consulted.	Methods: Search strategy; SDC 2
Search strategy	7	Present the full search strategies for all databases, registers and websites, including any filters and limits used.	Methods: Search strategy; SDC 2
Selection process	8	Specify the methods used to decide whether a study met the inclusion criteria of the review, including how many reviewers screened each record and each report retrieved, whether they worked independently, and if applicable, details of automation tools used in the process.	Methods: Screening
Data collection process	9	Specify the methods used to collect data from reports, including how many reviewers collected data from each report, whether they worked independently, any processes for obtaining or confirming data from study investigators, and if applicable, details of automation tools used in the process.	Methods: Data extraction
Data items	10a	List and define all outcomes for which data were sought. Specify whether all results compatible with each outcome domain in each study were sought and, if not, the methods used to decide which results to collect.	Methods: Data extraction; Synthesis and analysis
	10b	List and define all other variables for which data were sought. Describe any assumptions made about missing or unclear information.	Methods: Data extraction; Table 1; SDC 6
Study risk of bias	11	Specify the methods used to assess risk of bias in the included studies, including details of the tool(s) used, how many reviewers assessed each study and whether they worked independently.	Methods: Methodological quality

Section and Topic	Item #	Checklist item	Location where item is reported
assessment			of studies; Risk of Bias Within Studies; SDC 4
Effect measures	12	Specify for each outcome the effect measure(s) used in the synthesis or presentation of results.	Methods: Synthesis and analysis; Table 2
Synthesis methods	13a	Describe the processes used to decide which studies were eligible for each synthesis.	Methods: Synthesis and analysis
	13b	Describe any methods required to prepare the data for presentation or synthesis, such as handling of missing summary statistics or data conversions.	Methods: Data extraction; Synthesis and analysis
	13c	Describe any methods used to tabulate or visually display results of individual studies and syntheses.	Methods: Synthesis and analysis; Tables 1–3; Figure 2; SDC 6
	13d	Describe any methods used to synthesize results and provide a rationale for the choice(s). If meta-analysis was performed, describe the model(s), method(s) used to identify statistical heterogeneity, and software package(s) used.	Methods: Synthesis and analysis
	13e	Describe any methods used to explore possible causes of heterogeneity among study results.	Not formally explored by subgroup analysis or meta-regression; heterogeneity was quantified using I^2 and discussed qualitatively in Discussion and Limitations
	13f	Describe any sensitivity analyses conducted to assess robustness of the synthesized results.	Not performed
Reporting bias assessment	14	Describe any methods used to assess risk of bias due to missing results in a synthesis.	Not formally assessed
Certainty assessment	15	Describe any methods used to assess certainty or confidence in the body of evidence for an outcome.	Methods: Level of evidence; SDC 4a
RESULTS			
Study selection	16a	Describe the results of the search and selection process, ideally using a flow diagram.	Results; Figure 1
Study	16b	Cite studies that might appear to meet the inclusion criteria but were excluded, and explain why they were	Results; Figure 1; SDC 5

Section and Topic	Item #	Checklist item	Location where item is reported
selection		excluded.	
Study characteristics	17	Cite each included study and present its characteristics.	Results: Study Characteristics; Table 1; SDC 6a
Risk of bias in studies	18	Present assessments of risk of bias for each included study.	Results: Risk of Bias Within Studies; SDC 4
Results of individual studies	19	For all outcomes, present, for each study, summary statistics for each group and an effect estimate with precision where appropriate.	Results; Tables 2–3; Figure 2; SDC 6
Results of syntheses	20a	For each synthesis, briefly summarise the characteristics and risk of bias among contributing studies.	Results: Study Characteristics; Risk of Bias Within Studies; Mortality; Functional Outcomes
Results of syntheses	20b	Present results of all statistical syntheses conducted, including summary estimate, precision, heterogeneity, and direction of effect.	Results: Mortality; Table 2; Figure 2
Results of syntheses	20c	Present results of all investigations of possible causes of heterogeneity among study results.	Not formally assessed; discussed qualitatively in Discussion and Limitations
Results of syntheses	20d	Present results of all sensitivity analyses conducted to assess robustness of the synthesized results.	Not performed
Reporting biases	21	Present assessments of risk of bias due to missing results for each synthesis assessed.	Not formally assessed
Certainty of evidence	22	Present assessments of certainty or confidence in the body of evidence for each outcome assessed.	Results: Risk of Bias Within Studies; SDC 4a
DISCUSSION			
Discussion	23a	Provide a general interpretation of the results in the context of other evidence.	Discussion
Discussion	23b	Discuss any limitations of the evidence included in the review.	Discussion: Limitations
Discussion	23c	Discuss any limitations of the review processes used.	Discussion: Limitations
Discussion	23d	Discuss implications of the results for practice, policy, and future research.	Discussion: Implications for Practice and Research

Section and Topic	Item #	Checklist item	Location where item is reported
OTHER INFORMATION			
Registration and protocol	24a	Provide registration information for the review, including register name and registration number, or state that the review was not registered.	Methods; PROSPERO CRD42025643607
Registration and protocol	24b	Indicate where the review protocol can be accessed, or state that a protocol was not prepared.	Methods; PROSPERO CRD42025643607
Registration and protocol	24c	Describe and explain any amendments to information provided at registration or in the protocol.	Methods; no major amendments reported
Support	25	Describe sources of financial or non-financial support for the review, and the role of the funders or sponsors in the review.	Statements and declarations
Competing interests	26	Declare any competing interests of review authors.	Statements and declarations
Availability of data, code and other materials	27	Report which data, code, and other materials are publicly available and where they can be found.	Statements and declarations; Supplemental Digital Content
